# Benchmarking of real-time, field-deployable whole-genome sequencing of Plasmodium falciparum using Nanopore technology

**DOI:** 10.1099/mgen.0.001776

**Published:** 2026-07-29

**Authors:** Zahra Razook, Somya Mehra, Myo T. Naung, Brittany Gilchrist, Sachintha Wijegunasekara, Digjaya Utama, Dulcie Lautu-Gumal, Abebe A. Fola, Didier Menard, James Kazura, Moses Laman, Ivo Mueller, Leanne J Robinson, Melanie Bahlo, Alyssa E. Barry

**Affiliations:** 1 Population Health and Immunity Division, The Walter and Eliza Hall Institute of Medical Research, Melbourne, Victoria, Australia; 2 Life Sciences Discipline, Burnet Institute, Melbourne, Victoria, Australia; 3 Centre for Innovation in Infectious Disease and Immunology Research (CIIDIR), Institute for Mental and Physical Health and Clinical Translation (IMPACT), School of Medicine, Deakin University, Geelong, Victoria, Australia; 4 Department of Medical Biology, University of Melbourne, Melbourne, Victoria, Australia; 5 Vector Borne Diseases Unit, Papua New Guinea Institute of Medical Research, Madang, Madang Province, Papua New Guinea; 6 Department of Parasites and Insect Vectors, Institut Pasteur Paris, Paris, France; 7 INSERM U1201, Paris, France; 8 Department of Pathology, School of Medicine, Case Western Reserve University, Cleveland, USA

**Keywords:** drug resistance, genomics, long read, malaria, Nanopore, *Plasmodium falciparum*, structural variants

## Abstract

Malaria parasite genomes have been generated predominantly using Illumina short-read sequencing that requires expensive equipment, is time-consuming with complex protocols, and does not adequately interrogate complex genomic regions that harbour important malaria virulence determinants. The portable Oxford Nanopore Technologies MinION platform generates long reads in real time and may overcome these limitations. We present compelling evidence that Nanopore sequencing delivers valuable additional information for malaria parasites with similar data fidelity for single nucleotide variant (SNV) calls compared to standard Illumina whole-genome sequencing. We demonstrate this through sequencing of pure *Plasmodium falciparum* DNA, mock infections and natural isolates from low-density, asymptomatic infections. Nanopore has low error rates for haploid SNV genotyping and identifies structural variants not detected with short reads. Nanopore genomes can be directly compared to publicly available genomes and produce high-quality end-to-end chromosome assemblies including complex, previously difficult-to-access regions. Nanopore sequencing could expedite whole-genome surveillance of malaria and provide new insights into parasite genome biology.

**Impact Statement**

Genomic sequencing of pathogens is rapidly becoming a cornerstone of infectious disease surveillance, providing valuable information for disease control programmes. Malaria genomic surveillance has been rapidly expanding with endemic countries increasingly utilizing Nanopore platforms, which are an economical, portable and relatively easy-to-use technology that produces data from samples in real time with a sequencing approach that may uncover blind spots of the genome. Here, we present extensive benchmarking of Nanopore sequencing for whole-genome sequencing of *Plasmodium falciparum*, the most deadly and common malaria parasite in highly endemic areas. We developed an end-to-end workflow for high-quality data and showed that Nanopore performs similarly to gold standard methods and can reveal notoriously difficult-to-access parts of the genome and resolve entire chromosomes. This demonstrates the utility of Nanopore for whole-genome sequencing that could provide greater insights than existing sequencing approaches for use in research and public health.

## Data Summary

The authors confirm that all supporting data, code and protocols have been provided within the article or through supplementary data files.

## Introduction

Whole-genome sequencing (WGS) provides complete information about pathogens that along with epidemiological metadata can enhance control efforts [1] to track the spread of pathogens in real time [2], the emergence of drug resistance [3], responses to control interventions [4] and inform vaccine design [5]. Human malaria, a disease that has plagued humans for thousands of years, is caused by infection with *Plasmodium* species. It remains one of the world’s most widespread and deadly infectious diseases [6]. A major roadblock to WGS of malaria parasite isolates has been overcome through methods to enrich trace amounts of parasite DNA from finger prick blood samples contaminated with large amounts of human DNA [7]. However, generation of WGS has traditionally been restricted to well-equipped laboratories that can maintain large, expensive sequencing platforms as well as advanced analytical pipelines and human resources required for data processing. As Illumina short-read WGS (srWGS) requires extensive human expertise in both library preparation and instrument support, researchers in malaria-endemic countries have generally needed to send samples to large genome centres for sequencing, resulting in significant time delays and loss of data custodianship. However, for genomic surveillance to inform malaria control and elimination in a timely manner, large numbers of genomes with spatially dense sampling and the rapid generation of high-quality data at low cost will be needed, and this is only feasible through sequencing in proximity to endemic areas.

The Oxford Nanopore Technologies (ONT, UK) MinION is a portable, pocket-sized device that operates through a USB port in a personal computer. Nanopore sequencing involves the movement of DNA through a synthetic porous membrane (flow cell), resulting in unique ionic currents for each 6 bp window that are then translated into nucleotides in real time. The MinION device is an attractive option for malaria genomic surveillance because it can be deployed to field or clinical settings with minimal capital cost and training and thus provides potential for rapid genomic profiling [8]. In the clinical setting, MinION provides a platform to rapidly screen for multigene haplotypes associated with drug resistance [9, 10] and may provide a pathway for personalized treatment in some well-resourced settings. Long-read WGS (lrWGS) using MinION could be used to rapidly track the origins of outbreaks, as has been the case for other human pathogens [2, 11]. The low cost, relative portability and ease of use compared to other platforms suggest that this platform could be used not only in public health but also as a genomic research and surveillance capacity building aid [12–14]. While targeted amplicon sequencing methods have been adopted widely, WGS data can be retained for future studies and can continue to be analysed for a variety of additional features as knowledge expands.

Unique features of the *Plasmodium falciparum* genome contribute to the challenge of WGS. The genome is AT-rich (81%), with extended tracts of repetitive, low-complexity DNA [15], particularly in subtelomeric regions. Hypervariable multigene families, including the *var*, *rifin* and *stevor* families, have been difficult to characterize with srWGS because divergent short reads cannot be reliably aligned to a reference genome, although specialized pipelines have been developed [16]. In addition, malaria infections are often multiclonal, especially in high transmission regions [17], and therefore, an added challenge for malaria population genomics is reconstructing these clonal haplotypes. LrWGS has the potential to overcome these limitations by spanning complex regions, to enable more accurate assembly and to differentiate clonal haplotypes within multiple infections.

We aimed to develop and optimize Nanopore WGS protocols for *P. falciparum* and to benchmark the resulting lrWGS against that obtained using the current gold standard Illumina srWGS. Initially, we tested the platform on pure *P. falciparum* DNA extracted from laboratory isolates and compared the data to publicly available reference genomes. We then optimized the sequencing protocol for natural human infections using mock infections comprising DNA from a reference *P. falciparum* strain spiked with human DNA. Finally, we performed shallow sequencing of field isolates from Papua New Guinea (PNG) and Cambodia, allowing key parameters for field sample sequencing to be determined. The resulting data was used to estimate baseline error rates, to quantify the accuracy of single nucleotide variant (SNV) genotypes, to obtain drug resistance profiles and to benchmark against publicly available Illumina srWGS data from the same countries. Deep sequencing of cultured strains allowed *de novo* assemblies. These high-quality reference genomes enable characterization of full-length *var* genes and mapping of large structural variants (SVs), which were not achievable with srWGS. While srWGS-based approaches exist for SV detection [18–21], these are less straightforward and likely less accurate than lrWGS for resolving SVs at complex loci such as subtelomeric gene families. Optimized sequencing protocols and pipelines are provided so that this approach can be implemented in other laboratories to permit sequencing in the field.

## Methods

### Parasite isolates

Pure *P. falciparum* genomic DNA was obtained from three culture-adapted isolates including the reference strain 3D7 (Malaria Research and Reference Reagent Resource Centre, Bei Resources, Catalogue No. MRA102G); BB12, a descendant of the IT strain [22, 23] (provided by S. Rogerson, University of Melbourne); and XHA, a culture-adapted isolate from PNG [24] (provided by Ivo Mueller, Walter and Eliza Hall Institute). Annotated genome assemblies are available for 3D7 [25] and IT [26]. *P. falciparum*-infected blood samples (*n*=33) were collected in cross-sectional surveys in PNG, where malaria transmission and parasite genetic diversity are high [27], and in Cambodia, where transmission is low [28] with high levels of multidrug resistance [29].

### DNA extraction, parasite DNA enrichment and library preparation

For cultured *P. falciparum* lines, DNA extraction was performed using the DNeasy Blood and Tissue Kit (Qiagen, Germany) according to the manufacturer’s instructions. Extracted DNA was purified and concentrated as described previously [30], with SPRIselect beads (Beckman Coulter, Australia) used in lieu of Ampure XP beads. Briefly, 0.45 vol re-suspended beads were added to 1 vol of sample and incubated at room temperature for 10 min. After incubation, the bead/DNA mixture was placed on a magnetic rack for 5 min. The supernatant was removed without disrupting the DNA-bound beads, and the beads were then washed twice with 200 µl of 80% ethanol. After incubating for an additional 10 min at room temperature, the bound DNA was eluted with 60 µl of 10 mM Tris buffer. The quantity and quality of extracted DNA were measured using a Qubit Fluorometer (Invitrogen Life Technologies, USA) and NanoDrop 2000 (Thermo Fisher, USA), respectively. Size distributions were then analysed using TapeStation (Agilent Technologies, USA). Based on these results, 0.2 pmol of DNA was processed with the 1D genomic sequencing kit SQK-LSK108 following the manufacturer’s instructions (ONT).

To optimize library preparation for field samples, mock infections were prepared to mimic 1% parasitaemia by spiking 2 ng (0.083 ng µl^−1^) of 3D7 parasite DNA with 73 ng (3.066/µl) of commercial human DNA in a total volume of 24 µl. This is ~6.35 parasites/human genome and equivalent to a 1% parasitaemia (Text S1, available in the online Supplementary Material). Both methylation-dependent digested (McrBC) and undigested mock infections were tested to verify the efficacy of the enrichment protocol (described in detail below).

Library preparation and barcoding were done using the 1D genomic sequencing kit and native barcodes following the manufacturer’s instructions (Cat No. SQK-LSK108, ONT). Here, we assessed various adjustments to the post-whole-genome amplification (WGA, see below) and end prep purification. Following WGA, we trialled two methods of purification: 2.5V ethanol precipitation and 1.8V SPRI beads. Following end prep, we further tested 0.45V and 1 V bead clean-up volumes using AMPURE XP beads (Cat No. A63880, Beckman Coulter). According to the above protocol, 500 ng of each WGA sample was barcoded with native barcodes (Cat No. EXP NBD103, ONT) and eluted in 22.5 µl of nuclease-free water. Sample concentrations were quantified using a Qubit fluorometer (Invitrogen Life Technologies), and molarity estimates derived from TapeStation fragment size distributions (Agilent Technologies) were used to guide equimolar pooling. From each barcoded sample, an equimolar amount was combined to yield a total of 200 fmol (1,000 ng in total for six samples) for adapter ligation prior to loading onto the flow cell.

The optimal protocol was found to involve: 1.8V SPRI bead purification after WGA and 0.45V bead purification following end prep (Fig. S1A). All field samples were subjected to this protocol. Three to four field samples were multiplexed for each run by indexing with the native barcoding kit (Cat No. EXP NBD103, ONT).

For field isolates, DNA was extracted from dried blood spots using the FavorPrep 96-Well Genomic DNA Extraction Kit (Favorgen, Taiwan). To reduce costs and minimize the amount of human DNA contamination in the sequencing output, we developed a novel potentially universal enrichment strategy that is dependent on low methylation of the *Plasmodium* genome (less than 1%) [31, 32], relative to the high levels of methylation in the human genome (60–90%) [33]. The protocol involves two steps: a restriction digest that targets methylated cytosines followed by WGA using the Phi DNA polymerase primed with random hexamers. Briefly, 6 µl of DNA was subjected to restriction digestion with McrBC (New England Biolabs, USA) at 37 °C for 1 h and halted by incubation at 80 °C for 20 min. Gel electrophoresis confirmed a smear for human DNA alone, indicating that human DNA had been digested and maintenance of high-molecular-weight parasite DNA. Then, 2 µl of each field sample was subjected to WGA using the Illustra Genomiphi V2 DNA amplification kit (GE Healthcare Life Sciences, Australia) for 2 h at 30 °C followed by heat inactivation at 65 °C for 10 min. Amplified DNA samples were then purified using 1.8V bead purification (Beckman Coulter) and further treated with T7 endonuclease I to remove branch structures produced during WGA. All samples were amplified in duplicate-triplicate and pooled prior to library preparation.

To assess the efficacy of enrichment for mock and natural infections, we performed duplex quantitative real-time PCR (qPCR), targeting the *P. falciparum* 18S rRNA genes [34] and the human Plat1 gene [35]. We combined 0.2 µl (150 nM) of both forward and reverse primers, 0.45 µl (350 nM) of TaqMan probes, 6.5 µl of TaqMan Fast Advanced Mastermix (Applied Biosystem, USA) and 4 µl of target DNA, comprising of undiluted original DNA or a 1:100 dilution of the amplified product. Details of the primers and probes are presented in Table S1. To quantify parasite and human copy numbers, standard curves were generated by preparing tenfold dilution series of plasmid DNA containing parasite 18S rRNA gene [36] and from human genomic DNA (Cat No. G304, Promega, Australia). Thermal cycling was performed on a Light Cycler 480 II (Roche, Switzerland). The reaction volume was heated to 95° for 15 min, followed by 40 cycles of 95° for 15 s and 60° for 1 min.

### Nanopore sequencing and raw data analysis

Sequencing runs, each spanning 48 h, were performed using Nanopore flowcells MIN106/MIN107 (R9.4 and R9.5) and MinKNOW software V1.7.10-1.11.5 (ONT). Basecalling and demultiplexing were performed using the GPU-based Guppy V6 or Dorado basecallers as compatible high-accuracy models were not available for all samples within a single basecaller at the time of analysis; full details of basecaller assignments per sample are provided in Table S1. Both callers were run using the appropriate high-accuracy model for each flow cell chemistry to obtain reads in fastq format. Read length and quality summaries were subsequently generated with NanoPlot V0.16.4 [37]. Trimmed reads were then aligned to the two reference sequences using Minimap2 V2.4 [38] with the map-ont preset: (i) *P. falciparum* 3D7 (V3) [25] reference genome, used for SNV calling, variant genotyping, coverage statistics and *de novo* assembly polishing, and (ii) a concatenated *P. falciparum* 3D7, human HG19 and *Plasmodium vivax* P01 reference [39], used to estimate the proportion of parasite and human DNA in field isolates and to assess sequencing enrichment efficiency. Samtools V1.7 [40] utilities were used to sort and index and the resultant alignments to obtain sorted bam files. Coverage statistics and the proportions of parasite and human DNA were determined using samtools depth and several in-house helper scripts. Locus-wise alignment summaries were generated using samtools mpileup V1.7 [40], using a base quality threshold of 7. Alignment summaries were previously used to call variants using the bcftools multiallelic-caller V1.8 [41], in both genotyping and discovery modes; however, this approach has since been removed from the pipeline (Fig. S1B). Variant calling is now performed using the long-read-specific Longshot V1.0.0 variant caller [42], which leverages haplotype information from single-molecule sequencing reads to reduce false heterozygosity – an advantage over deep-learning-based callers that are extensively trained on diploid genomes and may be prone to higher rates of false heterozygous calls. Bcftools multiallelic-caller output was thus retained solely for baseline error rate calculations. Haplotypes were phased using read-depth (DP-tag from vcf file), and only the major allele was used for the downstream analysis. Indels were removed using vcftools V0.1.13 [43], and functional annotation of the resultant SNPs was performed using SnpEff V4.1l [44]. Quality filtration based on the depth of coverage at each locus and the proportion of reads supporting the called allele was performed using an in-house script (Text S2). A schematic of this pipeline for processing Nanopore data is shown in Fig. S1B.

Statistical analysis to determine associations between sample parameters and Nanopore sequencing output was done using the R package ggpubr [45]. Linear regression lines, with 95% confidence intervals (CIs), were generated with the function ggscatter, and Pearson’s correlation coefficient was computed with the stat_cor utility.

### Illumina sequencing and raw data analysis

We also performed Illumina sequencing of cultured *P. falciparum* isolates 3D7 (unknown origin), BB12 (Brazil) and XHA (PNG), which contain only *Plasmodium* spp. DNA, in addition to three Cambodian field isolates, which are contaminated with human DNA. These samples were used to benchmark MinION sequencing against the widely used Illumina sequencing approach. Library preparation for field isolates required a preliminary parasite DNA enrichment step (see above). Sequencing was performed as per the TruSeq Nano DNA sample preparation protocol (Illumina Inc., USA). Briefly, 200 ng of DNA was subjected to shearing, end-repair, A-tailing and adapter ligation, followed by enrichment with 15 cycles of PCR using a Bio-Rad T100 Thermal Cycler (Australia). The mean insert size was analysed using TapeStation (Agilent Technologies) with D1000 Screen Tape (Cat No. 5067-5582). Libraries were then sequenced using the NextSeq 500 platform (Illumina Inc.), generating 75 bp paired-end reads with a six-base index read. Data analysis was done using an in-house pipeline (http://github.com/bahlolab/pf_variant_calling_pipeline) in accordance with GATK best practices [46] (Text S3).

### Data analysis

#### Baseline error rates

To quantify false discovery rates (FDR) for *de novo* SNP characterization, variant calling was performed in discovery mode for both Nanopore and Illumina data to detect novel (haploid) SNPs in our cultured 3D7 isolate, relative to the available 3D7 reference genome (V3) [25, 47]. Sequencing data for both Nanopore and Illumina comprised mock infections (3D7 spiked with human DNA). For Nanopore WGS, we retained SNPs annotated as PASS from the Longshot V1.0.0 caller [42] with a minimum depth of coverage of 5× and genotyping quality above 20. Heterozygous positions are further resolved based on read depth using a custom script, considering only the allele supported by most reads (>50%). Illumina SNPs were filtered in accordance with GATK best practices (that is, QD≥2, MQ≥40, FS≤60, SOR≤3, MQRankSum≥−12.5 and ReadPosRankSum≥−8). To avoid detecting true variation between our laboratory isolate and the isogenic reference strain that may have arisen due to mitotic recombination in vitro [48], we considered only coding SNVs in (i) a panel of essential genes that would have been unlikely to differ between the cultured and reference strains (Text S4) and (ii) a set of high confidence SNVs obtained from an in-house reanalysis of MalariaGEN Pf3k plus PNG genomes [49].

#### Accuracy of SNV genotypes

Validation of SNV genotyping was done by first comparing *P. falciparum* isolates sequenced in-house using both Nanopore and Illumina platforms. Variants were called at 742,365 validated SNV loci obtained from an in-house reanalysis of the MalariaGEN Pf3k dataset (release 5), pooled with an additional set of 149 field isolates from PNG [49]. Discordance rates between Nanopore and Illumina genotypes for laboratory isolates 3D7, BB12 and XHA and the two Cambodian field isolates were quantified using an in-house script. Error rates in homopolymeric stretches more than 6 bp in length, identified using GATK VariantAnnotator (V3.5) were compared against non-homopolymeric stretches. Filtration parameters for haploid Nanopore SNV genotyping (depth of coverage at least 2×, 75% of reads supporting the called allele) were subsequently deduced.

Drug resistance profiling was then performed for field isolates with at least 1× coverage in 65% of the genome using the in-house custom pipeline (Fig. S1B). Genotypes were then screened for known resistance-associated variants in multiple genes including *crt* (PF3D7_0709000), *mdr1* (PF3D7_0523000), *dhfr* (PF3D7_0417200), *dhps* (PF3D7_0810800) and *kelch13* (PF3D7_1344700).

To determine whether there were any platform-specific effects in the SNV genotypes called from WGS data, we then performed a principal coordinates analysis (PCoA) of in-house Nanopore field isolate data (PNG) and Illumina field isolate data obtained from the MalariaGEN Pf3k/PNG dataset (Asia Pacific including PNG and Cambodia) based on 51,421 high-quality polymorphic SNVs that were present in those countries (Text S5).

#### *De novo* genome assembly

Raw Nanopore reads for BB12 and XHA were first assembled using Flye V2.9.3-b1797 [50] using default parameters without the scaffolding option. Ten iterations of consensus polishing were then performed with Racon V1.5.0 [51], which used the mapping of uncorrected reads to the scaffold, computed using minmap2 V2.4 [38] to generate consensus sequences. Draft ONT assemblies were further polished with neural network-based Medaka V1.11.3 [52]. Consensus assemblies constructed from Nanopore data only were also polished with Illumina sequencing data. Illumina reads processed with Trim Galore V0.4.4 [53] were first mapped against draft assemblies using bwa mem V0.7.13 [54]. Individual base errors, indels, block substitution events, gaps, local misassemblies and ambiguous bases in the draft assemblies were then corrected using Pilon V1.22 [55] to obtain hybrid Nanopore–Illumina assemblies. Illumina-only assembly was done with SPAdes V4.0.0. Assemblies are evaluated with QUAST. A schematic of this pipeline is shown in Fig. S1C. Draft assemblies for BB12 were benchmarked against its ancestral reference strain IT (Text S6).

Assemblies were compared against *P. falciparum* reference genomes using nucmer (MUMmer, V3.1 [56]), configured to identify one-to-one mappings between *de novo* contigs and reference chromosomes. Assemblies were first evaluated with BUSCO V5.

Gene annotation was assessed with the Companion pipeline (July 2024 web server version) [57]. BRAKER3 was then used for ORF prediction and annotations with the protein-coding file from Alveolata [58]. This pipeline contains an automatic training and gene prediction based on *ab initio* approach using Augustus [58]. eggNOG-mapper [59] and InterProScan [60] were used for functional annotation under default parameters. To improve consistency and biological interpretability, gene names and product descriptions were assigned based on sequence similarity to the *P. falciparum* 3D7 reference genome (PlasmoDB-68) using blast. The resulting annotations were then merged and harmonized with structural features, generating the final GFF3 annotation file. Coding sequence annotations that were shorter than 150 bp (as suggested by the NCBI) were removed from the final GFF3. Domain classification of annotated *var* gene candidates was performed using the VarDom 1.0 server [61] (Text S7).

#### SV calling

To assess the utility of Nanopore lrWGS for the characterization of SVs in *P. falciparum*, we performed a comparison of three distinct SV-calling pipelines for laboratory isolates BB12 and XHA: (i) short-read SV calling with GRIDSS [62], which combines split-read, read-pair and assembly approaches; (ii) long-read SV calling with Sniffles (default parameters) [63], which employs a split-read approach using input bam files mapped to 3D7 genome with minimap2; and (iii) SV detection through direct comparison of our *de novo* assemblies and 3D7 reference genome using command line version of Assemblytics [64] (Text S8). The rationale for using GRIDSS as a benchmark for short-read SV calling was twofold: in addition to combining a number of common approaches to short-read SV calling, GRIDSS was found to provide high precision across a range of SV types in a recent evaluation of over 60 short-read SV detection algorithms [65]. Concordance rates across the three methodologies were then computed (Text S8, Table S2). Systematic indel errors in Nanopore basecalling can lead to an overrepresentation of small deletions in low complexity and homopolymers; hence, we considered only SVs with length at least 200 bp, in line with general recommendations for Sniffles [63]. Several spuriously large SVs, spanning almost entire chromosomes in some cases, were detected by both GRIDSS and Sniffles. Hence, only SVs with length below 300,000 bp were retained. Due to low sequencing coverage, field isolates were not used for SV calling.

## Results

### Nanopore sequencing of *P. falciparum*

**Fig. 1.**
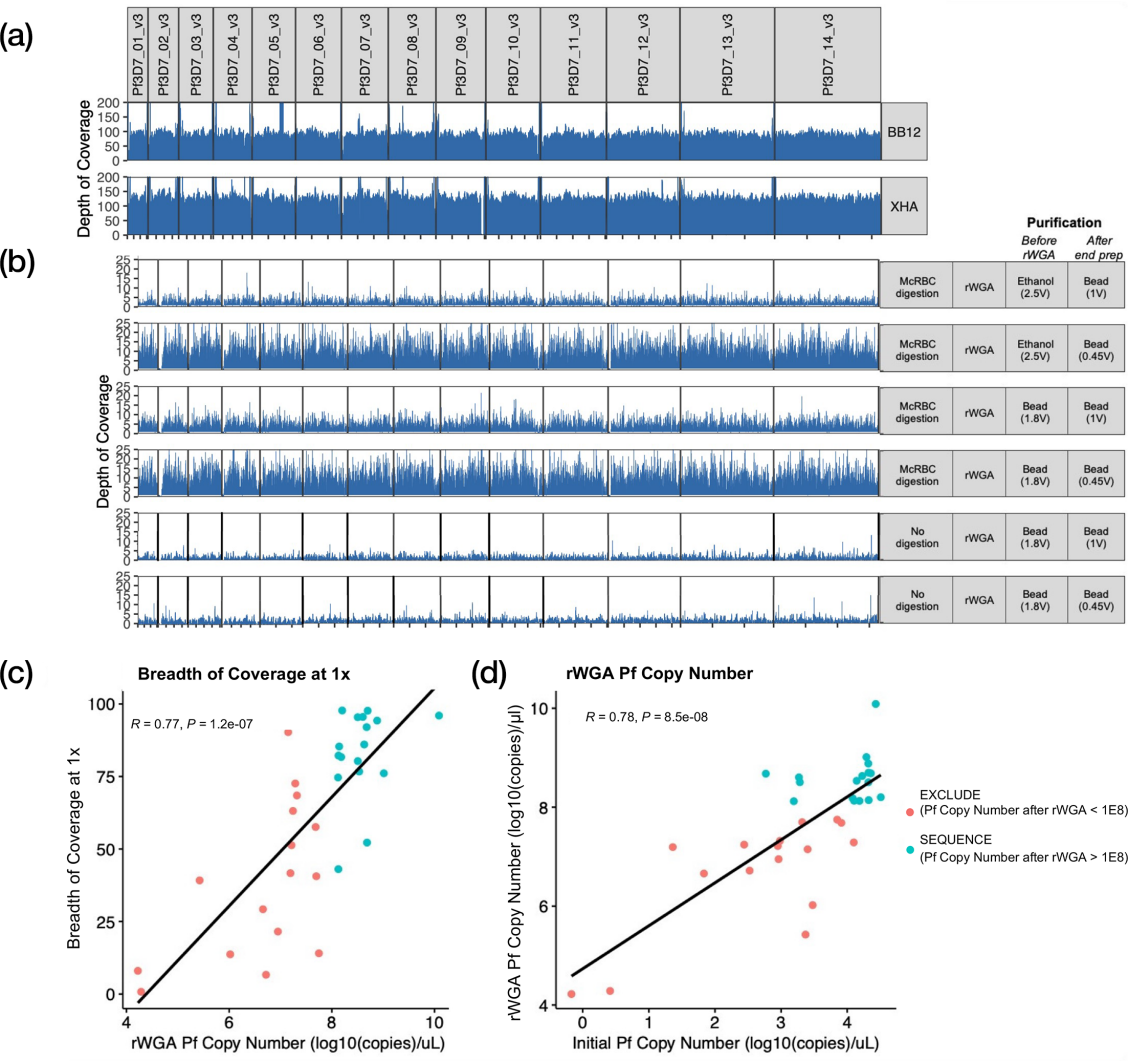
Output of MinION sequencing runs. (**a**) Schematic of genome coverage assessed in 5 kb bins of the parasite genome for pure *P. falciparum* laboratory isolates XHA and BB12 (pure parasite DNA). High uniformity, breadth and depth of coverage were attained for both laboratory isolates BB12 and XHA. (**b**) Optimization of parasite DNA amplification and library preparation using mock infections. Samples comprising 1% *P*. *falciparum* DNA using the laboratory isolate 3D7 were spiked with human DNA and subject to different enrichment and purification processes (rWGA with and without digestion with McrBC, with different bead clean-up conditions). The depth of sequencing coverage across the parasite genome is shown for each condition. Sequencing was run in multiplex for the six conditions. V represents volume. For instance, 1.8V indicates that we used 1.8 times the volume of bead or ethanol compared to the sample volume. (**c**) Impact of enriched parasite DNA density on sequencing success for all field isolates. Log-transformed post-enrichment *P. falciparum* density (18S rRNA copy number) is most strongly correlated with the breadth of coverage at 1× (the proportion of the genome covered by at least one read). A *P. falciparum* density of 2.5×10² copies/µl is the most appropriate diagnostic for a minimum breadth of coverage of 75% at 1× based on a receiver-operator characteristic analysis (Fig. S3). (**d**) Impact of pre-enrichment parasite DNA density on enrichment success for all field isolates. Data points are coloured based on whether they met the criteria for exclusion (red) or sequencing (green).

To test the capability of Nanopore for WGS of *P. falciparum*, test runs were first performed using pure *P. falciparum* DNA from the laboratory isolates XHA [24] and BB12 (a descendant of the Brazilian IT isolate) [22, 23] with each run on an independent flow cell for the full 48 h recommended (Fig. S1, Tables S3 and S4A). The total output of each run was 3.42 and 2.07 Gb, with N50 read lengths of 14.63 and 15.26 kb (mean read lengths of 8.80 and 6.95 kb) and maximum read lengths of 261.32 and 157.32 kb, respectively (Table S2A), with quality scores of more than Q5 for over 90% of reads. While this threshold is low relative to Illumina short-read sequencing (typically Q30), it reflects the quality range characteristic of the early R9 Nanopore flow cell chemistry used in this study and was selected to maximize data retention at sufficient coverage depth, particularly for field isolates sequenced at low depth. The two samples produced 120× and 76× mean depth of coverage (reads), with 95% of the genome covered by more than 30 reads and uniform genome-wide coverage (Fig. 1a).

*P. falciparum* field isolates obtained by collecting the blood of human volunteers contain substantial amounts of contaminating human DNA after DNA extraction [66]. Mock infections of 1% parasitaemia (20,000 parasites/μl [67]) made by spiking 3D7 DNA with human DNA were subjected to different enrichment conditions. The resulting six samples were run in multiplex on a single 48 h run, noting that the multiplexing enables economical production of ‘skim sequencing’ data, allowing the different conditions to be compared. Our novel enrichment procedure [McrBC digest plus random WGA (rWGA)] significantly increased the breadth and depth of parasite genome coverage (Figs 1b and S2, Table S4B) and the proportion of parasite DNA (≥83%) compared to rWGA alone (≤5%). After end repair, the 0.45V bead clean-up was associated with higher genome-wide read coverage than 1V bead clean-up. However, both 2.5V ethanol precipitation and 1.8V SPRISelect bead purification prior to library preparation performed similarly in terms of the read quality and output (Table S4B). Total bases generated may have been influenced by the longer median read length in the optimal bead clean-up combination (Table S2B). Median read lengths for mock samples were shorter than those observed for laboratory isolates sequenced without enrichment (Table S2B), which likely reflects DNA fragmentation introduced during the McrBC digestion and limited strand lengths generated during rWGA.

Using the optimized Nanopore sequencing protocol (1.8V bead purification after enrichment and 0.45V beads purification after end prep), we then sequenced 33 *P*. *falciparum* field isolates, multiplexing three to four samples per 48 h run (Table S4C). Field samples ranged in starting parasite densities from 0.674 to 32,200 parasite copies/μl as measured by qPCR (Q1=916 copies/μl, median=3000 copies/μl and Q3=16,840 copies/μl). Methylation-dependent McrBC digestion and rWGA resulted in between 114- and 810,000-fold enrichment (Q1=7940, median=21,200 and Q3=52,900). The proportion of reads mapping to the parasite genome varied between 0 and 85% (Q1=6%, median=18% and Q3=36%). Median read lengths ranged from 990 to 2,790 bp.

Key parameters associated with sequencing quality for individual field isolates are shown in Fig. 1(c, d), and Table S4 contains a range of input and output metrics. After enrichment, parasite density (parasite copies per microlitre adjusted from 18S rRNA copy number per microlitre) exhibited a strong correlation with the breadth of genome wide coverage at 1× (*R*=0.77, *P*=1.2E-7) (Fig. 1c). In a receiver operating characteristic curve analysis of field isolates, a parasite density threshold of 2.5×10^2^ is recommended as a practical guide for predicting sequencing success, defined as isolates achieving at least 75% breadth of coverage at 1× depth (Area Under the Curve =0.92, 95% CI: 0.81–10.00) (Fig. S3). This threshold provided the highest combined sensitivity and specificity in this dataset, though samples below this density are not excluded and may still yield adequate coverage. A comparison of copy numbers before and after enrichment (Fig. 1d) suggests that DNA samples with a minimum parasite density of 1,000 copies/μl should be selected for enrichment.

Stratification by flow cell showed a trend between the sequencing output and the amount of DNA loaded into a flowcell (Fig. S4). A decreasing trend between the total number of bases sequenced and the amount of DNA loaded was evident above 600 ng (121 fmol). Similarly, the proportion of reads with quality scores above 10 decreased as the amount of DNA loaded into a flowcell increased. During our experiments, we used R9.4 flow cells, which did not specify a loading threshold in the manufacturer’s protocol but recommended a minimum recovery of ~200 ng (40 fmol) of DNA after the adapter ligation step for an 8 kb sequencing library. The later R9.4.1 flow cell release provided guidance for a loading range of 5–50 fmol (~50–250 ng for an 8 kb library). Our findings align with these updated guidelines, showing that loading more than 600 ng (121 fmol) of DNA can cause pore clogging and decrease sequencing efficiency. Hence, clogged pores were more likely when higher amounts of DNA (>600 ng, 121 fmol) were loaded into the flowcell.

### Baseline error rates in Nanopore and Illumina sequencing

To quantify baseline error rates for Nanopore and Illumina, we measured FDRs at a given set of SNV loci by comparing Nanopore and Illumina 3D7 WGS genotype calls to those in the publicly available *P. falciparum* 3D7 reference genome (V3) [25, 47]. Analyses were restricted to high-confidence SNVs to ensure comparability with existing population genomic resources; indels and subtelomeric variants were excluded as they are not well represented in reference variant catalogues. Another possible complication is that while the core *P. falciparum* genome is stable in long-term *in vitro* culture, mitotic recombination can drive variation in antigen genes and subtelomeric regions [48]. We therefore restricted the reference genome-based analyses to SNVs situated in the core genome, with published 3D7 reference alleles to be treated as truth calls and alternate alleles found in the new sequencing data as baseline error, which are sequencing artefacts that could occur in the reference sequence (less likely) or the new data. We focused on two key use cases for SNV calling: *de novo* SNV discovery (e.g. to interrogate novel variants) and SNV genotyping at a set of known loci (e.g. to conduct population genomic analyses or drug resistance profiling).

#### SNV discovery

First, we considered FDRs in the new Nanopore and Illumina-derived 3D7 genotypes for the detection of *de novo* SNVs. We focused on reference alleles in coding sequences of 1,356 ‘housekeeping’ genes essential for *in vitro* asexual blood stage development [68], with a cumulative transcript length of 2,792,980 bp. Illumina was highly accurate with no coding SNVs in these genes relative to the reference genome, while Nanopore sequencing data gave rise to only seven coding SNVs after quality filtration (i.e. SNVs with a minimum depth of coverage of 5×, and genotyping quality above 20), with no SNVs retained when the genotyping quality threshold is increased to above 30 at a minimum depth of coverage of 5×. These results suggest a slightly higher FDR for *de novo* SNV discovery using Nanopore data (one sequencing artefact per 397,544 bp, FDR=0.00025%) relative to Illumina (0%), though it remains almost negligibly low. Albeit low coverage will also lead to lower power to detect novel variants. FDRs are contingent on coverage and variant filtration parameters – unlike the Illumina-specific GATK pipeline, which considers well-validated metrics to filter out poor alignments, best practice workflows are yet to be developed for Nanopore SNV genotyping.

#### SNV genotyping

If data is being generated for population genomic studies, genotyping by resequencing a validated set of polymorphic loci can be used to avoid error due to sequencing artefacts. To test for FDR in this case, we analysed the Nanopore and Illumina-derived 3D7 WGS at a set of 742,635 validated SNV loci. These SNV loci were previously identified by an in-house reanalysis of the MalariaGEN Pf3k release plus an additional set of 149 field isolates from PNG comprising 2,661 field isolates sampled from 15 malaria-endemic countries [49]. Again, this involved comparing the new 3D7 genotypes against the published 3D7 genome but only at the validated SNV loci. Of 684,737 SNVs successfully genotyped by both platforms, Illumina incorrectly called 13 alternate alleles, while Nanopore incorrectly called 18 alternate alleles after minimal filtration (i.e. minimum depth of coverage of 2× and with genotype quality of 20 at least 75% of reads supporting the called allele). Baseline error rates for genotyping SNVs at validated loci are therefore similar for Nanopore and Illumina and appear to be very low across both platforms (FDR<0.006%). However, since we mapped reads against an isogenic reference genome, read alignments are likely to be correct and higher genotyping accuracy may be driven by reference bias. It is therefore important to perform additional benchmarking of Nanopore and Illumina genotypes for isolates for which there is no isogenic reference genome.

### Accuracy of Nanopore genotypes

WGS analyses of *P. falciparum* commonly utilize high-quality SNV calls to conduct population genomic analyses [69]. Analyses were therefore restricted to high-confidence SNVs to ensure comparability with existing population genomic resources; indels and subtelomeric variants were excluded as they are not well represented in reference variant catalogues. To validate the genotyping accuracy and high coverage relative to low-coverage WGS, we performed a comparison of reference (3D7) versus alternate (non-3D7) allele calls for Nanopore and Illumina SNV genotypes derived from laboratory strains BB12 (Brazil) and XHA (PNG) with very high coverage (>70×) and two Cambodian field isolates with low-coverage Cam_01 and Cam_02 (<5×). Nanopore and Illumina data from these isolates was aligned to the 3D7 reference genome (V3), and variants were called. We then compared haploid (dominant) allele calls in Nanopore- and Illumina-based genotypes. This analysis investigated all variant calls obtained for the 742,635 high-quality variants described above [49, 69].

Nanopore SNV calls exhibited strong bias towards reference allele calls at very low depths of coverage seen in field isolates where the percentage of correct Nanopore alternate calls is lower than that for reference calls for all isolates (Table 1, reference allele >99%; alternate allele=64.59–94.30% before filtering). At low depth (Cam_01, Cam_02), alternate alleles in Illumina were more likely to be called as reference alleles by the Nanopore analysis pipeline, suggestive of conservative allele calls (>71.19% for Cam_01 and 64.59% for Cam_02, relative to 92.71% for XHA and 94.30% for BB12). Reference allele calling bias, which arises during read mapping, has previously been reported for Nanopore SNV genotyping [70], with implications for haplotype reconstruction due to the overrepresentation of reference haplotypes [71].

**Table 1.** Comparison of haploid Nanopore and Illumina genotypes at 742,635 high-quality loci Genotype calls were classified as concordant when both platforms supported the same allele state, either the reference allele (REF; the allele present in the 3D7 reference genome) or the alternate allele (ALT; a non-3D7 reference allele). Calls were classified as discordant when the platforms supported different alleles, including REF/ALT, ALT/REF or different ALT alleles. Concordance rates were calculated using loci with valid calls in both platforms.

Sample: XHA					Sample: BB12				
	Illumina (60×)	Nanopore (117×)	Unfiltered	Filtered		Illumina (31×)	Nanopore (84×)	Unfiltered	Filtered
Concordant	REF	REF	683,648	677,524	Concordant	REF	REF	656,151	650,085
	ALT	ALT	7,906	6,963		ALT	ALT	8,301	8,725
Discordant	REF	ALT	612	336	Discordant	REF	ALT	489	224
	ALT	REF	1,133	180		ALT	REF	776	217
	ALT 1	ALT 2	10	10		ALT 1	ALT 2	13	8
Missing	n/a	n/a	49,056	57,352	Missing	n/a	n/a	76,635	83,106
Percentage of correct Nanopore REF calls:			99.83	99.97	Percentage of correct Nanopore REF calls:			99.88	99.97
Percentage of correct Nanopore ALT calls:			92.71	95.27	Percentage of correct Nanopore ALT calls:			94.30	96.88
Overall concordance rate:			99.75	99.92	Overall concordance rate:			99.81	99.93
**Sample: Cam_01**					**Sample: Cam_02**				
	Illumina (4.5×)	Nanopore (4.8×)	Unfiltered	Filtered		Illumina (3×)	Nanopore (2.7×)	Unfiltered	Filtered
Concordant	REF	REF	453,740	304,211	Concordant	REF	REF	331,371	170,256
	ALT	ALT	3,156	2,518		ALT	ALT	1,831	1,296
Discordant	REF	ALT	1,243	322	Discordant	REF	ALT	981	171
	ALT	REF	2,412	208		ALT	REF	2,225	122
	ALT 1	ALT 2	34	26		ALT 1	ALT 2	23	12
Missing	n/a	n/a	281,780	435,080	Missing	n/a	n/a	405,934	570,508
Percentage of correct Nanopore REF calls:			99.47%	99.93%	Percentage of correct Nanopore REF calls:			99.33%	99.93%
Percentage of correct Nanopore ALT calls:			71.19%	87.86%	Percentage of correct Nanopore ALT calls:			64.59%	87.63%
Overall concordance rate:			99.20%	99.82%	Overall concordance rate:			99.04%	99.82%

**Fig. 2.**
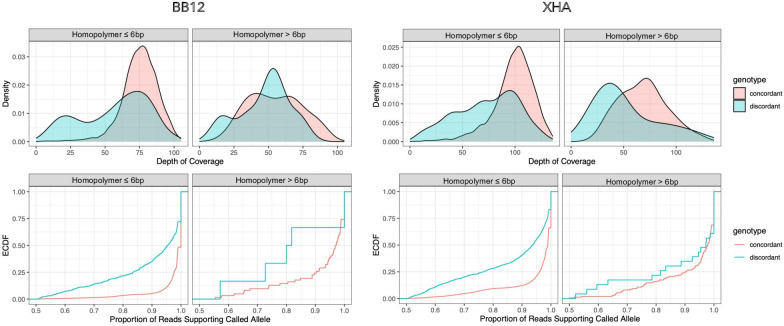
Variant quality metrics for discordant and concordant Nanopore genotype calls in both long homopolymeric (>6 bp) and short/non-homopolymeric (≤6 bp) regions for laboratory isolates: (**a**) BB12 and (**b**) XHA. Density plots show distributions for the depth of coverage by locus. Calls are labelled concordant or discordant based on agreement with Illumina genotypes at the same locus. Density plots show read depth distributions by locus. Empirical cumulative distribution functions (ECDFs) show distributions for the proportion of reads supporting the called allele. Left-shifted curves indicate lower support for detected alleles. In both homopolymeric and non-homopolymeric regions, discordant calls frequently had a smaller proportion of reads supporting the called allele than concordant calls.

Since Nanopore sequencing using R9 flow cells has been shown to exhibit significant systematic error in homopolymeric regions [72], we also stratified the allele calls in laboratory isolates XHA and BB12 described above based on genomic content, by differentiating between calls within long homopolymeric tracts of length greater than 6 bp and those in non-homopolymeric regions with no homopolymer or those less than or equal to 6 bp. Discordance rates with Illumina were observed in both long homopolymeric tracts (>6 bp) and short homopolymeric regions (≤6 bp), particularly at low coverage. However, the alignment quality around discordant MinION calls in long homopolymeric tracts was often poor, with multiple genotypes being called, slightly lower Nanopore read depth than in flanking genomic regions (Fig. 2). This causes fewer reads to align with the called allele, leading to reduced coverage. In non-homopolymeric regions, discordant calls and concordant calls between Nanopore and Illumina exhibited similar signatures. Discordant calls between Nanopore and Illumina could thus be explained by low coverage and a low proportion of read mapping to the called allele (that is, false heterozygosity) (Fig. 2). Poor alignments of Nanopore sequence data around homopolymer tracts [72] and false heterozygosity due to Nanopore sequencing error, particularly at low depths of coverage [73], have been previously documented.

As a strategy to remove these artefacts, SNV loci were then filtered by the depth of coverage and the proportion of reads supporting the allele call; metrics that previous studies have also adopted [9]. Excluding SNV loci with read support across multiple alleles does not account for heterozygosity or multiclonality. However, this approach was deemed appropriate since the laboratory isolates were presumed to be monoclonal, and while shallow sequencing of field isolates generated inadequate coverage to detect minor clones, dominant clones will be genotyped. Therefore, for the field isolates, we only retained those genotypes with a minimum depth of coverage of 2× where at least 75% of reads supported the called genotype (i.e. if 2× coverage, both reads must support the call).

After these filtering steps, overall discordance rates between haploid Nanopore and Illumina genotypes were consistently low, even for the Cambodian field isolates. While these isolates had a large proportion of missing loci after filtering, a large number of genotyped loci remained to enable high-resolution population genomic analysis (XHA: 304,211 loci, BB12: 170,256 loci, Table 1). Of note, filtered Nanopore reference calls had above 99.93% concordance with Illumina for all four isolates, with the laboratory isolates only slightly higher (99.97%, Table 1). As for the unfiltered allele calls, alternate allele calls were more discordant after filtration, with 88% supported by Illumina for Cam_01 (4.8× coverage) and Cam_02 (2.7× coverage) and over 95% for laboratory isolates BB12 (117× coverage) and XHA (84× coverage). These results indicate that Nanopore alternate allele calls are less reliable at low depths of coverage. As expected, discordance rates in homopolymeric tracts greater than 6 bp were found to be substantially higher than overall discordance rates (Table S5), highlighting the need to exercise caution in analysis of calls from these regions.

Random errors in Nanopore sequencing were mitigated by increasing read coverage, as evidenced by the lower discordance rate for laboratory isolates with high coverage. However, the persistence of genotyping error at high depths of coverage points to systematic differences between the two platforms. We therefore attempted to identify characteristic features of concordant and discordant calls that passed quality filtration. The relative frequencies of concordant and discordant genotype calls after filtration were stratified by the variation type at discordant sites found within Nanopore and Illumina data. The most common discordant genotypes across the trialled isolates were the Illumina (treated as truth calls) → Nanopore (treated as erroneous calls) transitions G → A and C → T, followed by the transversions T → A and A → T (Fig. S5). In general, transition errors between similarly structured bases (i.e. between two-ring purines or one-ring pyrimidines) were more pronounced than transversion errors for Nanopore sequencing, which might reflect errors in the basecalling algorithm. Given that Nanopore calls are generated based on minute changes in current as the DNA passes through the pore, it is not surprising that biochemically similar bases are harder to distinguish and result in erroneous calls.

### Drug resistance profiling

As a measure of Nanopore’s ability to generate functionally relevant information including from low-read depth sequencing of asymptomatic field isolates, we determined SNV genotypes for laboratory isolates XHA and BB12, 1 Cambodian field isolate (Cam_01, 4.8×) and 15 PNG field isolates that met quality thresholds (Tables 2 and S4). Genotypes were obtained for a range of known drug resistance marker loci including *crt* (chloroquine, amodiaquine, piperaquine), *dhfr-ts* (pyrimethamine), *dhps* (sulfadoxine), *mdr1* (lumefantrine, mefloquine) and *kelch13* (artemisinin). Only genotype calls with depth of coverage at least 2× and at least 75% of reads supporting the called allele were retained (filtered calls). Functional annotations were applied to filtered variants, allowing the extraction of both nucleotide and amino acid haplotypes.

**Table 2.** Summary statistics for *de novo* assemblies of *P. falciparum* genomes

	BB12 (IT ref)	XHA (3D7 ref)
No. of contigs	17	19
Total length (bp)	23,120,614	23,128,735
Longest contig (bp)	3,374,248	3,280,578
GC content (%)	19.44	19.49
BUSCO (completeness)*	C: 98.7% [S: 98.2%, D: 0.5%] F: 0.2%, M: 1.1%, *n*: 3,642, E: 0.8%	C: 98.8% [S: 98.8%, D: 0.0%] F: 0.1%, M: 1.1%, *n*: 3642, E:1.9%

*C, complete BUSCOs; S, complete and single-copy BUSCOs; D, complete and duplicated BUSCOs; F, fragmented BUSCOs; M, missing BUSCOs; *n*, number of benchmarking universal single-copy orthologues (BUSCOs); E, internal stop codons.

**Fig. 3.**
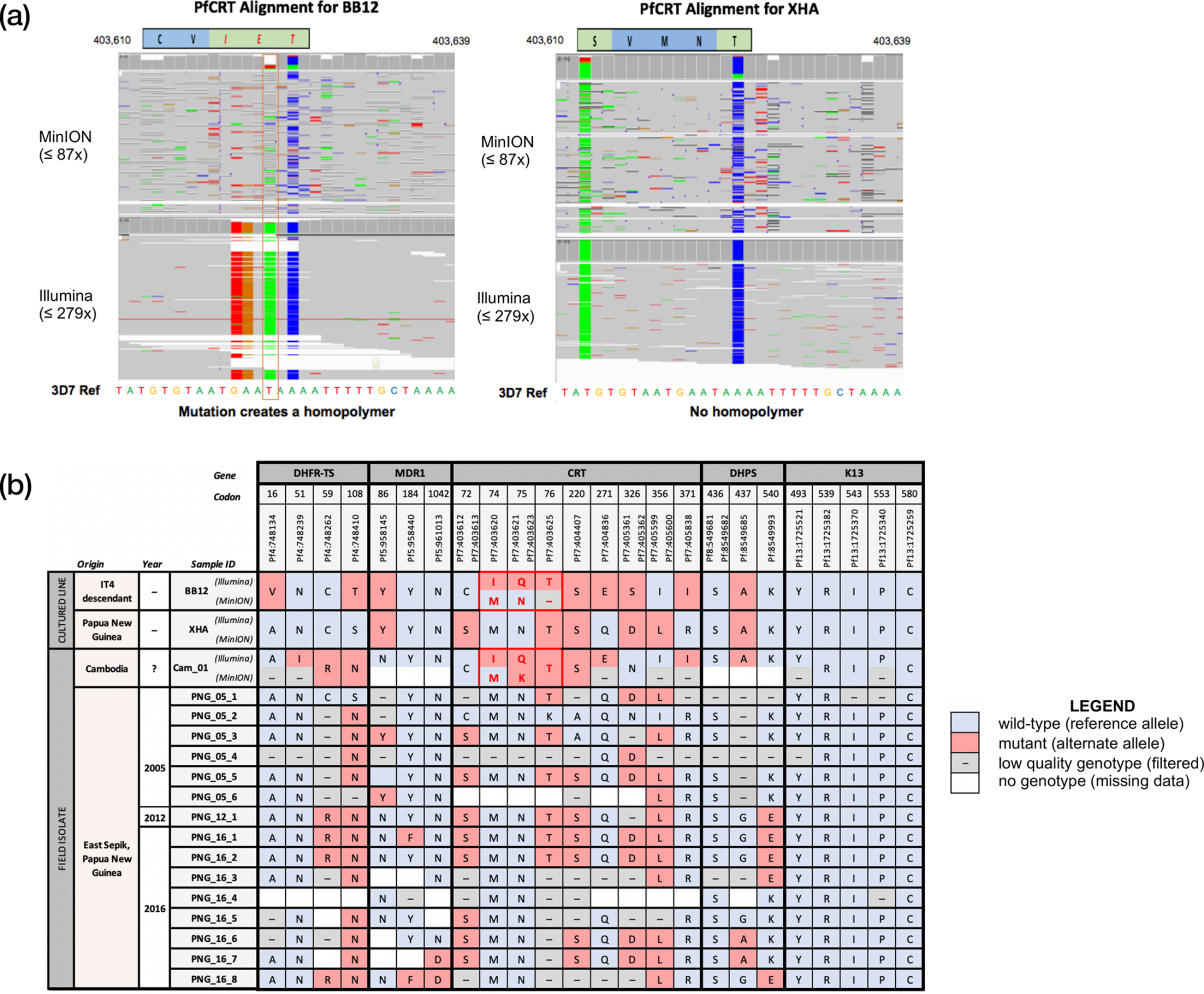
Drug resistance profiling. (**a**) Alignments of Nanopore sequence data across *crt* codons 72 to 76 for laboratory isolates BB12 and XHA. Nanopore and Illumina genotypes are discordant for BB12, where the mutation at position Pf3D7_07_v3:403625 has given rise to a homopolymeric tract. Nanopore and Illumina genotypes are concordant for XHA, for which there is no such homopolymeric stretch. (**b**) Drug resistance amino acid haplotypes for laboratory isolates XHA, BB12 and Cam_01 (both Nanopore and Illumina), and PNG field isolates with breadth of coverage >65% at 1× (Nanopore only). Isolates were genotyped at loci corresponding to key resistance-associated variants in genes *crt*, *mdr1*, *dhfr*, *dhps* and *k13*. Note that all CRT variants previously associated with resistance that were screened included T93S, H97Y, F145I, M343I and G353V; however, all isolates had wild-type alleles and thus are not shown. Red boxes indicate resistance-associated alleles, while wild-type alleles are indicated in blue. Missing genotypes (for which there was no sequence data) and low-quality genotypes (which were removed after quality filtration) are shown in white and grey, respectively.

Haplotypes generated from Nanopore and Illumina sequencing data for the laboratory isolates BB12 and XHA and field isolate Cam_01 were compared first. Filtered drug resistance gene haplotypes were generally concordant between the two sequencing platforms, except for *crt* codons 74 to 76. Here, for isolates BB12 and Cam_01, alternate alleles identified using Illumina data were called as reference alleles using Nanopore data (Fig. 3). For Cam_01, reference alignment bias due to low coverage may have contributed to the erroneous genotype call at *crt* codon 74; BB12, however, had high overall coverage. Inspection of the alignments in this region revealed that for BB12, one of the resistance-associated variants gave rise to a homopolymer. No such homopolymer was introduced by the XHA mutation (Fig. 3a), hence resulting in concordant haplotypes. Since Nanopore sequencing is subject to systematic error in homopolymeric stretches, this again demonstrates that caution should be exercised when potential homopolymeric tracts are encountered in loci selected for genotyping. However, we note that recent advances in Nanopore flow cell chemistry and improved basecalling algorithms will help mitigate these errors further.

Amino acid haplotypes spanning key resistance-associated variants across drug resistance-associated genes are presented in Fig. 3(b). Only Nanopore sequenced field isolates with a minimum breadth of coverage 65% at 1× are presented, and except for K13, only polymorphic loci within the dataset are shown. Some genotype calls were filtered due to low coverage. Missing genotype calls (occurring when no reads align to a particular locus) tend to be clustered, since genome-wide coverage for some Nanopore field isolates was uneven with no data captured for some genomic regions. These issues should be resolved as methods for parasite genomic DNA extraction and enrichment improve.

### Nanopore genotypes can be directly compared to publicly available *P. falciparum* Illumina data

Given that most publicly available *P. falciparum* field isolate genomes have been determined using Illumina, we asked whether genotypes obtained using Nanopore could be directly compared with these genotypes or whether Nanopore sequencing gave rise to platform-specific effects, thus limiting biological insight from direct comparisons. We examined clustering patterns for Nanopore-sequenced field isolates (originating from PNG or Cambodia) relative to Illumina-sequenced isolates (originating from PNG, Cambodia, Vietnam, Laos or Thailand).

This analysis was based on genotype calls at the 742,365 high-quality SNVs identified through our in-house reanalysis of the MalariaGEN P3k plus PNG dataset. Genotype missingness filtration by isolate (isolates with <30% missingness across SNVs were retained) and SNV loci (SNVs with <10% missingness across isolates) was performed to avoid possible biases introduced by imputation. Monomorphic loci were removed since they were uninformative; SNVs were also not filtered by minor allele frequency to avoid ascertainment bias. After filtration, we retained 10 new Nanopore- (all PNG field isolates) and 1,020 Illumina-sequenced field isolates from the MalariaGEN Pf3k plus PNG dataset, genotyped at 51,421 polymorphic SNV loci.

**Fig. 4.**
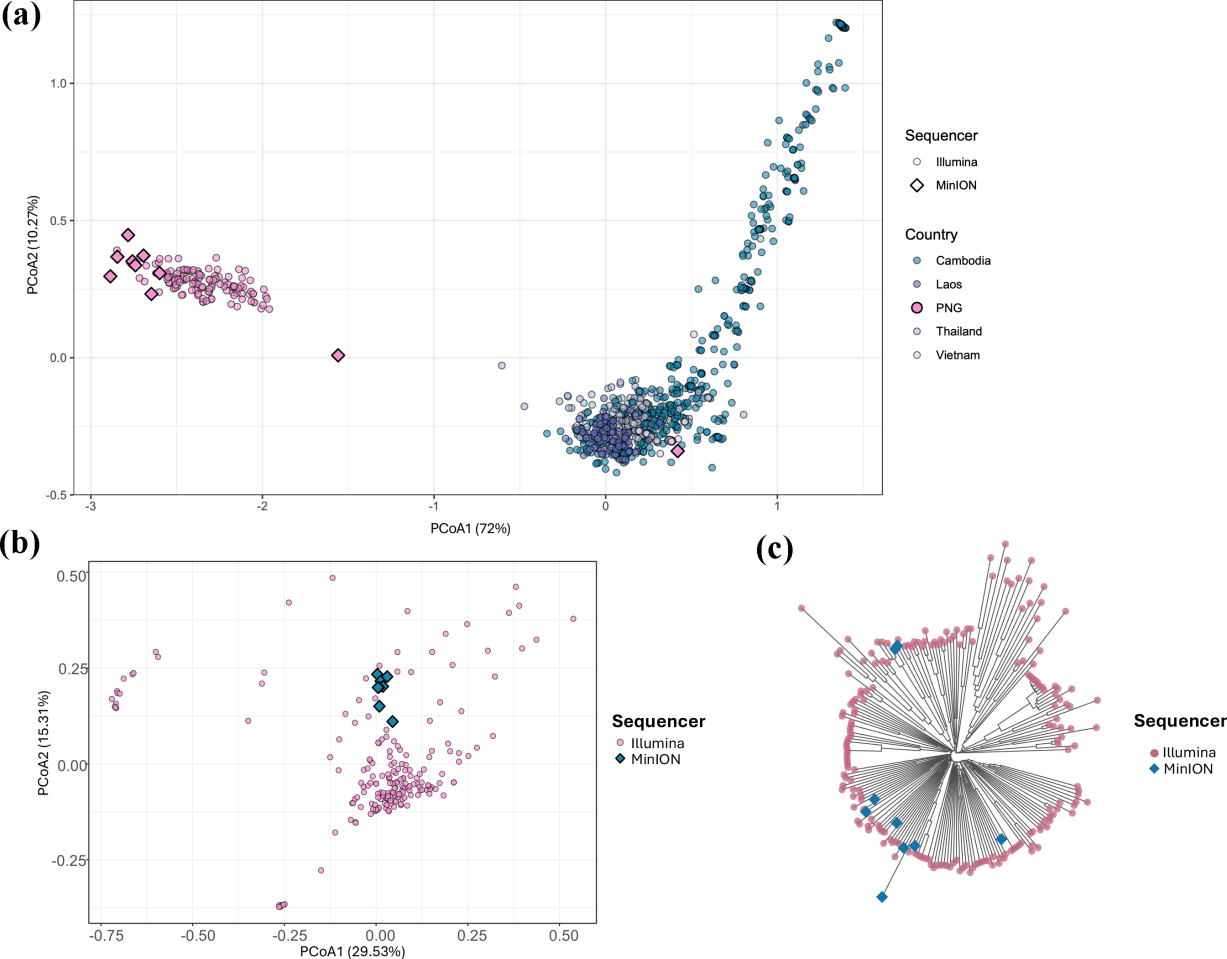
Clustering of Nanopore and Illumina-sequenced field isolate genomes from PNG and South-East Asia (Cambodia, Laos, Thailand and Vietnam). (**a**) PCoA was performed on the resultant distance matrix to obtain a 2D representation of the data. Each point represents a distinct isolate, with colours representing geographic origins and shapes representing sequencing platforms. Nanopore- and Illumina-sequenced field isolates originating from PNG cluster together closely in the PCoA except one sample. Due to low coverage or high missingness, only a subset of the Nanopore-sequenced isolates met the quality threshold for inclusion in this analysis. (**b**) PCoA of PNG-only genotypes. (**c**) Neighbour-joining tree of PNG-only genotypes constructed from the pairwise genetic distance matrix using the *ape* R package. Tip colours indicate sequencing platform.

PCoA was then performed on pairwise genetic distances between isolates, defined to be the proportion of successfully genotyped loci with shared alleles for each pair of isolates. Projections of isolates onto two dimensions are visualized in Fig. 4(a). Expected geographical clustering patterns emerged, with field isolates originating from PNG and Southeast Asia generally clustering distinctly. Nanopore- and Illumina-sequenced field isolates originating from PNG cluster together. As there is significant clustering observed between Asian and PNG isolates, we also evaluated PCoA-based and phylogenetic clustering using only the PNG genotypes. This demonstrates that apart from two isolates that fall between the Asian while the Nanopore isolates tend to cluster more closely together in the PCoA, they are unrelated and cluster throughout the phylogenetic tree, providing confidence that there are no systematic platform-based biases (Fig. 4b, c).

### *De novo* assembly of high-depth Nanopore WGS provides complete chromosome assemblies and allows characterization of highly polymorphic regions

*De novo* genome assembly (i.e. assembly of reads to each other, rather than a reference genome) can further improve characterization of parasite genomes, enabling the inclusion of highly polymorphic genomic regions that are typically blacklisted during variant calling due to extreme polymorphism relative to the reference genome. The advent of lrWGS has greatly enhanced the continuity and completeness of *de novo* genome assembly, particularly in low-complexity and repetitive genomic regions that have been difficult to resolve with short-read fragments (<1,000 bp) [74].

To characterize repetitive and highly polymorphic genomic regions that would map to the correct reference loci or not map at all due to high diversity (e.g. high density of variants), we generated *de novo* genome assemblies for the high-depth laboratory isolates XHA and BB12 by integrating both Nanopore and Illumina data. Initial long, contiguous scaffolds constructed using only Nanopore WGS using Flye demonstrated superior performance over Illumina WGS using SPAdes and was further refined by polishing using Racon and Medaka (Table S6). These scaffolds, though contiguous, might contain individual base errors, indels, block substitution events, gaps and local misassemblies. To refine them, we generated a hybrid assembly (Nanopore and Illumina) and applied a further polishing step, following common genome assembly workflows (https://github.com/nanoporetech/ont-assembly-polish) (Table S6). Though hybrid assemblies often provide slightly enhanced accuracy, the Nanopore-only assembly is still effective and yields similar results and the expected gene content provided by BUSCO scores is high (Tables 2 and S4).

The BB12 assembly was compared against the reference genome of its closest relative, the parent IT4 strain (V4) (Text S6). However, for XHA, which is an isolate from PNG, no close relative reference genome is available, so we compared the *de novo* assembly against the 3D7 reference genome; the 3D7 reference contains 14 complete nuclear chromosomes and has higher continuity than the IT4 reference genome. Assembly continuity for both BB12 and XHA was high, with all nuclear chromosomes except chromosome 5 spanned by a single contig (Fig. S6). Breakpoints for chromosome 5, which was spanned by two contigs in both assemblies, varied for XHA and BB12. Alignment of the BB12 assembly against the IT4 reference genome (V4) revealed that the four shortest contigs outside of core nuclear chromosomes from IT4 were unable to map to the BB12 assembly.

Although *de novo* contigs and reference chromosomes were generally concordant in core genomic regions, alignments in subtelomeric hypervariable regions were more fragmented. This fragmentation could reflect true variation between laboratory isolates and reference isolates (e.g. XHA vs 3D7) but may also be a marker of translocation and contig misassembly for homologous lines (e.g. BB12 vs IT4). A 150 kbp translocation from chromosome 13, for instance, is apparent in the downstream subtelomeric region of contig PfBB12_11.

**Fig. 5.**
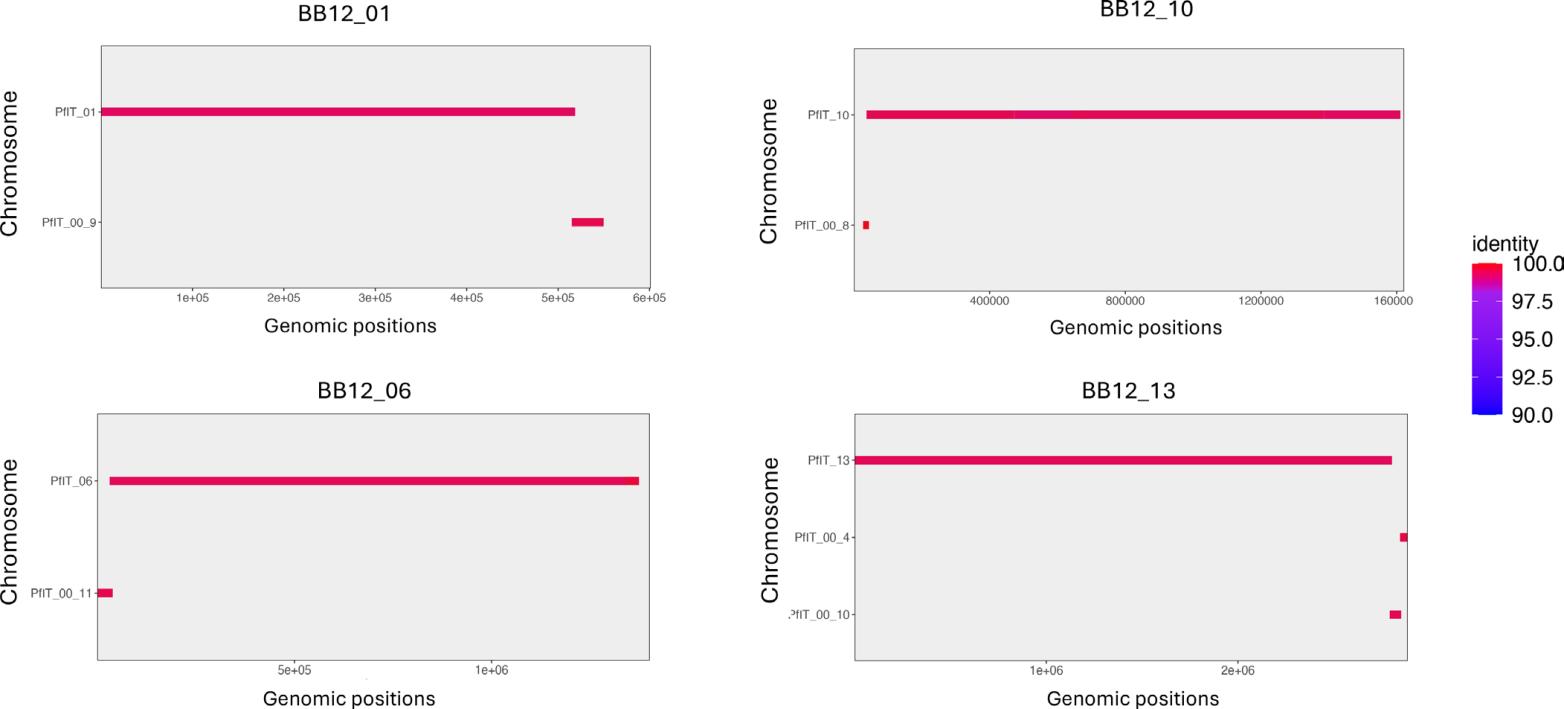
Enhanced continuity of Nanopore BB12 assembly, relative to the IT4 reference genome (V4). More complete assembly of chromosomes 1, 6, 10 and 13 was enabled through long-read sequencing. Fragment PfIT_00_9 has been localized to the subtelomeric region of PfIT_01, fragment PfIT_00_8 to the subtelomeric region of PfIT_10 and fragment PfIT_00_11 to the subtelomeric region of PfIT_06, while fragments PfIT_00_4 and PfIT_00_10 have been identified as neighbouring subtelomeric regions of chromosome 13. One-to-one mappings between *de novo* contigs and reference genomes have been computed using nucmer (MUMmer, V3.1 [56]).

The BB12 assembly allowed a more complete characterization of chromosomes 1, 6, 10 and 13 relative to the IT4 reference genome (V4). Since our BB12 assembly exhibited higher continuity than the IT4 assembly (V4), we were able to localize fragment PfIT_00_09 to the subtelomeric region of PfIT_01, fragment PfIT_00_11 to the subtelomeric region of PfIT_06 and fragment PfIT_00_8 to the subtelomeric region of PfIT_10, while fragments PfIT_00_4 and PfIT_00_10 were identified to be neighbouring subtelomeric flanks of chromosome PfIT_13 (Fig. 5).

A genome-wide summary of annotated features in our assemblies for XHA and BB12 is shown in Table 3, with corresponding statistics for the 3D7 reference genome shown, as a comparison. As expected, BB12 annotated results such as mean gene locus size are similar to the IT reference genome annotation. The elevated number of predicted coding genes compared to the 3D7 reference genome indicates that ORFs in the *de novo* assemblies may have been disrupted by mismatch and indel errors, which can be further refined using transcriptomic data. For example, some annotated genes were fragmented, with multiple neighbouring annotations likely corresponding to the same gene. Nevertheless, we were still able to characterize a large number of genomic features in our assemblies.

**Table 3.** Summary of annotated genomic features for *de novo* assemblies using Nanopore compared to the Illumina 3D7 and IT reference genomes

	BB12 assembly	XHA assembly	3D7 reference*	IT reference*
No. of genes	6,499	5,021	5,562	5,577
Gene density (genes/Mb)	281.4	217.2	237.8	245.00
No. of coding genes	6,104	4,813	5,318	5,035
No. of non-coding genes	395	208	244	409
Mean gene size (bp)	2,233	3,408	3,299	2,480
GC content (%)	19.49	19.44	19.34	19.35

*Ensembl protist database (July 2024).

As evidence of the ability of Nanopore lrWGS to characterize complex and highly polymorphic regions, we analysed the *var* genes encoding the major variant surface antigen, PfEMP1. We identified 27 complete, 16 split and 11 partial *var* genes for BB12 and 18 complete, 16 split and 15 partial *var* genes for XHA. Domain structures for all complete and partial *var* genes are shown in Fig. S7.

### Improved SV calling

**Fig. 6.**
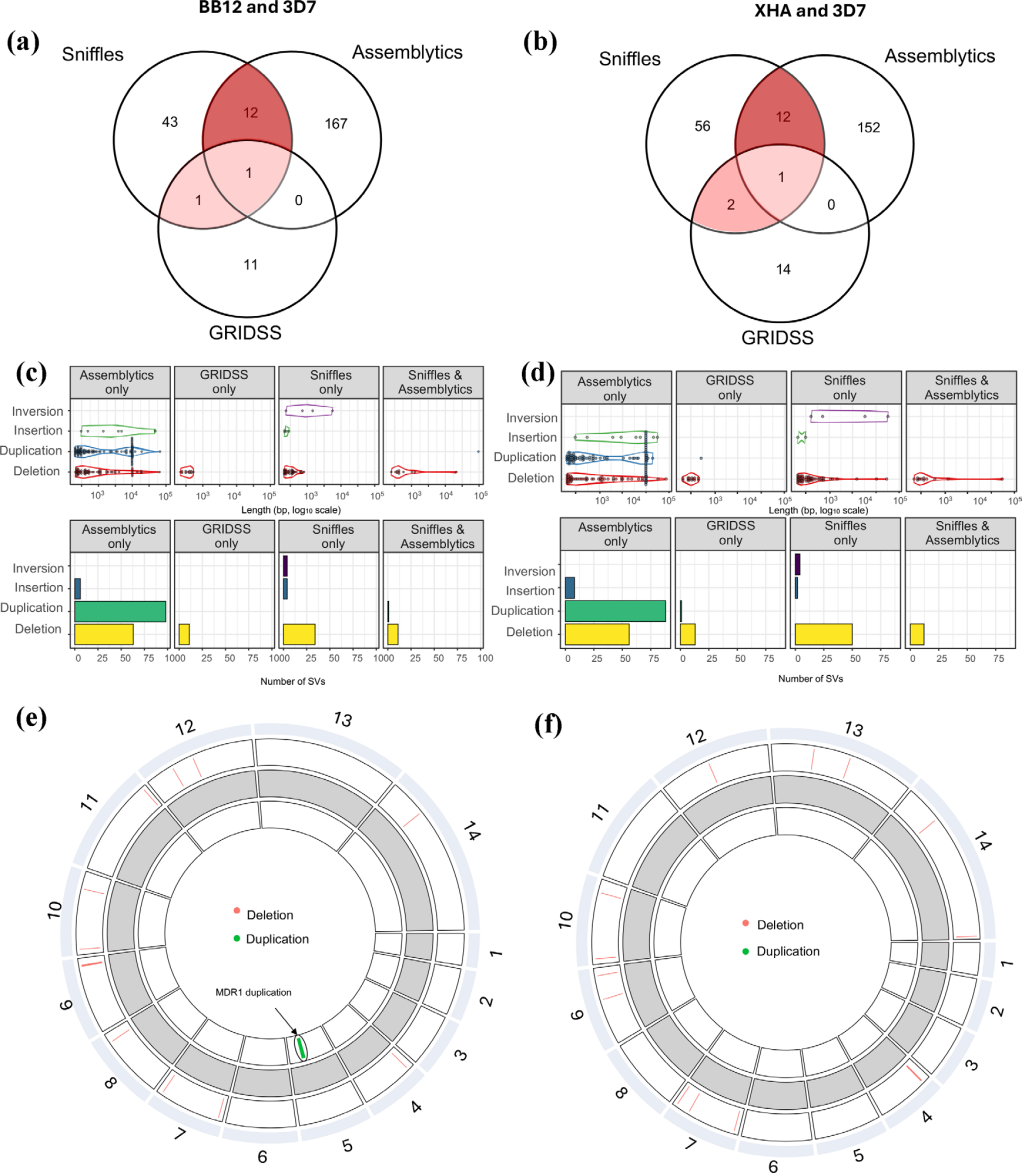
Comparison of SV calling pipelines using long reads only (Sniffles), short reads only (GRIDSS) and our *de novo* assemblies (Assemblytics). Overlaps in the sets of high-quality SVs identified by each pipeline, BB12 and XHA, are shown in (**a**) and (**b**), respectively, with red intensity indicating the number of overlaps. SVs identified by different pipelines are considered equivalent if they overlap by at least 1 bp, correspond to the same SV class and have similar lengths (i.e. the length of the shorter SV is at least 75% of that of the longer SV) (Text S8). Sniffles (which uses long reads only) and Assemblytics (which is informed by our *de novo* assemblies) show a reasonable number of concordances, while GRIDSS (which uses short reads only) exhibits very little concordance with either Sniffles or Assemblytics. SV types and lengths for BB12 and XHA are shown in (**c**) and (**d**), respectively. SV length and type distributions are generally quite similar for Sniffles and Assemblytics. For each panel, SVs are stratified into four categories: Assemblytics only, GRIDSS only, Sniffles only and SVs concordant between Sniffles and Assemblytics. Within each category, the upper violin plots show the distribution of SV lengths (x-axis: length in base pairs on a log₁₀ scale) for each SV type (deletion, duplication, insertion and inversion); the lower panel shows the total count of SVs (x-axis: number of SVs) for each SV type. SV length and type distributions are generally similar between Sniffles and Assemblytics. Genome-wide SV maps relative to 3D7 reference genome, restricted to SVs concordant across Sniffles and Assemblytics, are shown for isolates BB12 (**e**) and XHA (**f**), respectively. Both Sniffles and Assemblytics detected the known MDR1 duplication in BB12.

As the comparison of different SV callers is beyond the scope of this work, we did not include such analyses. While srWGS-based approaches can detect certain classes of SVs such as copy number variants (CNVs) using read-depth and breakpoint analyses (e.g. MalariaGEN Pf8 CNV calling pipeline [18], Manta [19], DELLY [20] and LUMPY [21]), these methods are less straightforward and likely less accurate for complex and repetitive loci compared to long-read sequencing [75]. LrWGS has emerged as a promising tool for SV detection, particularly complex SVs [26]. We conducted SV analysis only for high depth laboratory isolates XHA and BB12 and compared to the reference genome 3D7 V3.0 [25, 47]. As a first step, we compared concordance rates across three trialled SV pipelines focused on SVs longer than 200 bp to avoid systematic errors associated with Nanopore sequencing [63] (Fig. 6). SVs detected using long reads only (Sniffles [63]) and our *de novo* assemblies (Assemblytics [64]) exhibited reasonable levels of concordance with each other than with the short-read only pipeline (GRIDSS [62]), with 12 high-quality SVs shared between Sniffles and Assemblytics across both isolates (~30% of Jaccard overlap across the combined call sets of both pipelines). While most SVs remained unique to each pipeline, this level of inter-pipeline concordance is expected reflecting fundamental differences in detection approaches: Sniffles uses split-read alignment to a reference genome, while Assemblytics detects SVs through direct comparison of *de novo* assemblies to the reference. SVs detected using GRIDSS had very little concordance with either Sniffles or Assemblytics called SVs. Short-read SV calling (GRIDSS) identified fewer high-quality SVs longer than 200 bp, with most high-quality SVs within the length range 30–200 bp. Longer SVs failed to pass default quality filtration parameters. Complex SVs, such as inversions, are more sensitive to detection with long-read-based alignment approaches like Sniffles, aligning with observations from Liu *et al*. [76]. Distributions of SV types and lengths for the three pipelines are shown in Fig. 6(c, d).

We then screened high-quality SVs to identify functionally relevant variation. For BB12, a 96 kbp duplication on chromosome 5 spanning mdr1 (PF3D7_0523000) was identified by both Sniffles and Assemblytics (as well as manual inspection of the mdr1 gene in the above *de novo* assembly annotation step), but not GRIDSS. A qPCR assay confirmed the presence of the duplication spanning mdr1 (data not shown). The amplification of mdr1 has been associated with resistance to a range of antimalarial drugs, including artemisinin, lumefantrine, mefloquine and halofantrine [77]. While this SV was successfully captured by Nanopore lrWGS data, Illumina srWGS failed to identify this amplification. No gene amplifications were observed for other drug resistance markers. A random downsampling of BB12 fastq reads, followed by SV detection using the Sniffles method, indicates that at least 15× mapped read coverage is required to detect the *mdr1* duplication. This threshold can serve as a guideline for sequencing field isolates to ensure reliable SV detection.

The results demonstrate enhanced sensitivity and resolution of SV calling pipelines leveraging Nanopore lrWGS data, compared to approaches using srWGS data in isolation. Genome-wide SV maps for isolates BB12 and XHA, constructed using SVs concordant across Sniffles [63] (based on lrWGS mapping to a reference genome) and Assemblytics [64] (based on *de novo* assemblies), are shown in Fig. 6(e, f), respectively.

## Discussion

The Nanopore MinION sequencer is an attractive platform for sequencing malaria parasites in the field and laboratory with several new studies recently published [13, 14, 78–80]. However, Nanopore sequencing is known to have higher error rates in comparison to Illumina platforms. In addition, *P. falciparum* has an AT-rich genome, with a higher probability of homopolymers, known to be the cause of high error rates in Nanopore WGS [9, 25]. Here, we benchmarked Nanopore lrWGS against Illumina srWGS to reveal the utility of Nanopore sequencing for *P. falciparum* laboratory and field isolates. The results reveal a smaller baseline error rate than expected for haploid SNP genotyping using Nanopore long-read sequencing. Discordant allele calls between Nanopore and Illumina data were more frequent in homopolymer regions greater than 6 bp, which are known to have poor sequence quality using the R9 (R9.4 and R9.5) Nanopore flow cells used here, and are expected to decrease with R10 Nanopore pore designs commonly used at the current time. R10 flow cells now match Illumina’s sequencing accuracy, making them suitable for applications like filling gaps in genomes caused by homopolymers, insertions or deletions [81]. Using mock infections, we developed an optimized library preparation protocol for whole blood-derived DNA samples enriched for parasite DNA after treatment with McrBC digestion and rWGA, which could also be used for other *Plasmodium* spp. such as *P. vivax* and even other pathogens. We note that the enrichment workflow may introduce DNA fragmentation, resulting in shorter read lengths for field and mock samples compared to laboratory isolates sequenced without enrichment. This represents an inherent trade-off of the enrichment approach. Through sequencing field samples with a range of infection densities at low depth, we were able to provide recommendations for selecting *P. falciparum* isolates for Nanopore WGS. This dataset was generated using earlier Nanopore flow cells and kits; more recent approaches now achieve substantially improved coverage, comparable to current standards. Early runs resulted in outputs of 2–3 Gb, but at the time of writing, we are generating 20–30 Gb of data per run using the SQK-LSK109 and SQK-LSK114 kits (unpublished data), which would increase coverage by almost tenfold and therefore allow multiplexing of up to 12 samples. In addition, we report streamlined current state-of-the-art analytical pipelines for SNV and SV genotyping and *de novo* assembly, which yielded high-quality variants with low error rates and complete chromosomes including highly polymorphic subtelomeric regions and large SVs missed by srWGS.

As Illumina short-read sequencing is considered the gold standard WGS approach, we benchmarked the accuracy of Nanopore WGS against Illumina WGS using both the publicly available reference genomes and newly generated data from isogenic parasite isolates. Removing dominant sources of variation (namely, indels, SNVs in homopolymers, subtelomeric polymorphisms and heterozygosity) significantly increases the accuracy of genotyping with the Nanopore platform. We acknowledge that applying these filters reduces some of the added value of lrWGS – particularly its ability to resolve complex indels, subtelomeric gene families and SVs – which represent important targets for malaria surveillance. These variant classes are instead addressed through the *de novo* assembly analyses, which leverage the full capabilities of long-read data but require high depth of coverage. The low error rates (<1% off filtered calls) computed were based on haploid SNV genotyping and are significantly lower than reported error rates for diploid or ‘double haploid’ SNP genotyping, when heterozygous calls are taken into account [48]. In addition, transition-type errors (G to A or C to T) were more frequent than transversion-type errors, possibly reflecting errors in basecalling. Training basecalling models with taxon-specific data, such as the custom-Kp model trained on *K. pneumoniae* [82, 83] or the *Mucoplasma* taxon-specific custom-trained Bonito basecalling model [84], has been shown to significantly reduce such errors. These findings highlight the value of tailoring basecalling to a specific genomic context, particularly for species with lower GC content, such as *P. falciparum*. However, given the high quality and accuracy of the data produced from the basecallers used in this study, we have opted not to attempt this. Future research should explore the development of custom-trained basecalling models for *Plasmodium* species, leveraging advances in neural network architectures to further enhance accuracy and adapt to unique genomic features. While the accuracy of Nanopore reference calls was high, alternate allele calls were found to be less reliable at low depths of coverage; however, overall discordance rates remained low since reference calls far outnumber alternate calls. Reliably detecting alternate alleles at low depths of coverage may be problematic for Nanopore sequencing in some applications.

Using Nanopore data, drug resistance profiles were generated including SNVs identified in five marker genes. The results demonstrate the utility of the Nanopore MinION platform as a tool for drug-resistance profiling, even at very low depths of coverage (breadth of coverage ≥65% at 1×), granted caution is exercised around potential homopolymeric tracts flanking target loci where error rates were higher. High concordance with Illumina data was observed except for *crt* codons 72–76, which contain the chloroquine resistance haplotype. XHA (SVMNT) showed high concordance between Nanopore and Illumina data, but there were several discordant positions for BB12 (CVIET), due to a T to A mutation within codon 75 that creates a homopolymer; however, this may be somewhat overcome by R10 flow cells. As these haplotypes are more common in some parts of the world, caution is warranted when genotyping *crt* using Nanopore sequencing. Some drug resistance genes and samples were genotyped more consistently, e.g. *k13* was fully genotyped in almost all samples, while *crt* had more missing data due to the introduced homopolymer. With increasing information on the variant frequency in target populations, however, missing genotypes can be imputed [85] and flow cell chemistry has improved to allow more accurate sequencing of homopolymers. Despite missing data for some samples, a large number of PNG isolates from different time points were successfully genotyped for all genes. The results illustrate a high prevalence of resistance markers for chloroquine and antifolate drugs [86, 87] yet a complete lack of *k13* mutations associated with partial artemisinin resistance. This is critical information for PNG given the recent reports of a small number of *k13* C580Y mutant parasites in Wewak in 2016 [86], only 50 km from the collection site for the samples sequenced here and in other parts of PNG [88, 89]. The ability for Nanopore to return results within 48 h at field sites, rather than weeks or months after samples have often left the country of collection, means that field-relevant results can be returned in a time frame relevant for decision-making regarding local treatment regimens.

As platform technologies and associated software change and improve, it will be important to ensure that new data can be directly compared to other previously generated and public datasets [69], to track parasite evolution as they respond to changes in transmission and different selection pressures. While our analysis of discordance rates between Nanopore and Illumina (haploid) SNV genotyping suggests the presence of some systematic differences between the two sequencing platforms, these effects may not be of a sufficient magnitude to significantly skew population genomic analyses. Our results confirm the viability of population genomic analyses combining Nanopore and Illumina data. In addition, this project took place over several years as Nanopore reagents, flow cells and analysis solutions were rapidly updated and improved, and therefore, some of the methods we used to generate data have been replaced. However, the approaches for library preparation remain valid. Recently, we updated the analytical workflow, including implementing the Dorado basecaller and Longshot V1.0.0 variant detection and reanalysed the raw data.

We also demonstrate the ability of Nanopore lrWGS to resolve hypervariable and low complexity genomic regions that have been difficult to characterize with short reads. Nanopore data allowed the construction of highly contiguous genomic scaffolds that could then be polished with Illumina data to correct small-scale errors, including indels, individual base errors, block substitution events and local misassemblies. We generated highly complete and contiguous *de novo* assemblies, validated through a comparison of BB12 against its ancestral reference strain IT4 [22, 23, 26]. Although a number of *de novo* assemblies for *P. falciparum* drawing on single-molecule real-time (SMRT) lrWGS using the PacBIO platform have been published [26], we presented high-quality *de novo* genome assemblies for *P. falciparum* generated using Nanopore lrWGS.

While SVs play an important role in the genomic diversity of *P. falciparum* [90], shortcomings persist in the detection of SVs with short-read sequencing [75]. Long-read sequencing has the potential to enhance the sensitivity and specificity of SV detection; however, applications of lrWGS to characterize structural variation in the *P. falciparum* genome [91] have been limited. Studies have focused on comparisons between reference genomes and *de novo* genome assemblies generated from PacBIO SMRT long-read sequencing data. We demonstrated the enhanced ability of Nanopore long reads to capture SVs relative to short reads alone by comparing pipelines for SV detection relying on Illumina only mapped to a reference genome [62]; Nanopore only mapped to a reference genome [63], as well as *de novo* genome assemblies [26]. While other short-read variant callers may be better suited for detecting larger SVs, our analysis did not identify mdr1 amplification using GRIDSS with short-read data. However, this does not preclude the possibility that alternative short-read assemblers might perform differently. Notably, we detected a 96 kbp duplication on chromosome 5 spanning mdr1 in BB12 using both lrWGS (Sniffles) and *de novo* assembly (Assemblytics). The duplication of *mdr1*, which is associated with multidrug resistance [77], was confirmed by qPCR. While further research is required to generate robust sensitivity and specificity estimates for SV detection, Nanopore sequencing is a promising tool for fine-scale mapping of complex SVs in the *P. falciparum* genome.

Nanopore long-read sequencing using the MinION portable device has been promoted as a useful platform for real-time portable sequencing solutions, with applications to malaria surveillance [8]. Here, we have optimized Nanopore sequencing protocols and data analysis for high-quality *P. falciparum* WGS at a slightly higher cost (USD100) to that of Illumina (USD90). The results described offer insight into the quality and utility of Nanopore long-read sequencing and its inherent limitations. Following filtering of low-confidence variation, we observed acceptably low error rates and very high concordance of resulting genome-wide (haploid) SNP genotypes for both pure *P. falciparum* DNA and field isolates contaminated with high amounts of human DNA. While this work used R9 flow cells commonly used when these experiments were conducted, the recent wide-scale implementation of R10 flow cells will undoubtedly deliver even more accurate results, especially across homopolymers, and thus may enable the removal of this filtering step [92]. Long reads generated using this platform improve whole-genome assembly and the detection of hypervariable regions and SVs. Nanopore genomes are directly comparable to publicly available Illumina genomes and reveal novel insights of practical importance for malaria control programmes including population structure and drug resistance. Finally, the Nanopore sequencing platforms also offer the potential for research equality by enabling sequencing in country for the many countries still affected by malaria.

## Supplementary material

10.1099/mgen.0.001776 Supplementary Material 1.

## Abbreviations

CI: confidence interval
CNV: copy number variant
FDR: false discovery rate
lrWGS: long-read whole-genome sequencing
ONT: Oxford Nanopore Technologies
PCoA: principal coordinates analysis
PNG: Papua New Guinea
qPCR: quantitative real-time PCR
rWGA: random whole-genome amplification
SMRT: single-molecule real-time
SNV: single nucleotide variant
srWGS: short-read whole-genome sequencing
SV: structural variant
WGA: whole-genome amplification
WGS: whole-genome sequencing
