# Supplementary material for: Benchmarking of real-time, field-deployable whole-genome sequencing of Plasmodium falciparum using Nanopore technology
Source: Microb Genom. 2026 Jul 29;12(7):001776. doi: 10.1099/mgen.0.001776 (PMC13435551; doi:10.1099/mgen.0.001776)
Supplement: Supplementary file 1 [file mgen-12-01776-s001.pdf]

SUPPLEMENTARY MATERIALS

Razook, Mehra and Naung *et al*. Benchmarking of real time, field-deployable whole genome sequencing of *Plasmodium falciparum* using nanopore technology

SUPPLEMENTARY METHODS

Supplementary Text 1. Preparation of mock infections
Supplementary Text 2. Details of Nanopore SNV quality filtration
Supplementary Text 3. Pipeline for processing Illumina data
Supplementary Text 4. Validated SNV sets for baseline error rate analysis
Supplementary Text 5. Principal coordinates analysis (PCoA) of isolates from PNG and Southeast Asia
Supplementary Text 6. Benchmarking of BB12 *de novo* assemblies against the IT reference genome
Supplementary Text 7. Gene annotation and *var* gene domain classification
Supplementary Text 8. Structural variant calling pipelines

Supplementary Text 1: Preparation of mock infections
Mock infections were prepared to mimic ~1% parasitaemia by spiking 2 ng of *P. falciparum* 3D7 DNA (0.083 ng/μL) with 73 ng of commercial human DNA (3.066 ng/μL) in a total volume of 24 μL. Based on the estimated genome masses of *P. falciparum* (26 fg per haploid genome) and human DNA (6.1 pg per diploid genome), this mixture contained approximately 3192 parasite genomes and 503 human genomes per μL, corresponding to 6.35 parasite genomes per human genome. Assuming one diploid human genome per WBC and a blood composition of approximately 5000 RBCs per 8 WBCs, this ratio corresponds to 6.35 parasites per 625 RBCs, or approximately 10 infected erythrocytes per 1000 RBCs (6.35 × 1000/625 = 10.16). This is equivalent to a parasitaemia of ~1%.

Supplementary Text 2: Details of Nanopore SNV Quality Filtration
Nanopore genotype calls were assessed individually for each sample using two locus-wise quality metrics:

- The number of high-quality bases mapping to the locus ($\mathrm{CovDepth}$), and
- The proportion of high-quality reads supporting the called allele ($\mathrm{PropReads}$).

Both metrics were extracted from the DP4 tag, as described in the VCF file specification, which encodes the breakdown of high-quality bases mapping to the forward/reverse reference/non-reference alleles:

$$\mathrm{DP4} = \mathrm{REF_{forward}}, \mathrm{REF_{reverse}}, \mathrm{NREF_{forward}}, \mathrm{NREF_{reverse}},$$

where $\mathrm{(N)REF_{forward}}$ and $\mathrm{(N)REF_{reverse}}$ denote the number of high-quality bases mapping to the forward and reverse (non-)reference alleles respectively. The DP4 was parsed using string manipulation functions in GNU *awk,* and quality metrics were calculated using the following formulae:

$$\mathrm{CovDepth} = \mathrm{REF_{forward}} + \mathrm{REF_{reverse}} + \mathrm{NREF_{forward}} + \mathrm{NREF_{reverse}}$$

$$\mathrm{PropReads} = \begin{cases} \dfrac{\mathrm{REF_{forward}} + \mathrm{REF_{reverse}}}{\mathrm{REF_{forward}} + \mathrm{REF_{reverse}} + \mathrm{NREF_{forward}} + \mathrm{NREF_{reverse}}} & \text{if REF call} \\ \dfrac{\mathrm{NREF_{forward}} + \mathrm{NREF_{reverse}}}{\mathrm{REF_{forward}} + \mathrm{REF_{reverse}} + \mathrm{NREF_{forward}} + \mathrm{NREF_{reverse}}} & \text{if ALT call} \end{cases}$$

Since the DP4 tag considers only high-quality bases, $\mathrm{CovDepth}$ was lower than depth of coverage encoded in the DP tag for some loci. Here, CovDepth refers specifically to sequencing depth, defined as the number of high-quality bases covering a given position, rather than breadth of coverage, which describes the proportion of the target genome covered by reads.

Supplementary Text 3: Pipeline for Processing Illumina Data
Isolates sequenced in-house on the Illumina platform were analysed in accordance with GATK best practices[1]. Briefly, Illumina adapters were identified using *Picard Tools MarkIlluminaAdapters V2.17.3*[2] and reads were aligned against the P. *falciparum* 3D7 (version 3) reference genome using *bwa mem V0.7.13*[3]. Indels were realigned using the *GATK IndelRealigner V3.5.0*[4], and duplicate reads were identified with *PicardTools MarkDuplicates.* Base quality score recalibration was the performed using the *GATK BaseRecalibrator*. Haploid variants were called using the GATK *HaplotypeCaller* in both DISCOVERY mode (which outputted all detected variants) and GENOTYPE_GIVEN_ALLELES mode (which genotyped isolates at a specific set of loci).

Supplementary Text 4: Validated SNV sets for Baseline Error Rate Analysis
We generated two validated SNV sets to measure baseline error rate analysis in newly generated Nanopore and Illumina 3D7 laboratory isolate WGS to compare to the published 3D7 genome[27]. The SNV sets included reference variant calls from coding sequences in 1356 essential genes, and 742,635 genome-wide SNV loci derived from an in-house re-analysis of the WGS data from 2,668 *P. falciparum* clinical isolates including 2,512 isolates from 14 countries spanning Southeast Asia, Central Africa, West Africa, and Oceania sourced from the MalariaGEN Pf3k project release 5.1, and an additional 156 isolates from Papua New Guinea obtained from the MalariaGEN *P. falciparum*

Community Project [25,26]. All isolates were sequenced using Illumina short-read technology and reads were aligned to the 3D7 reference genome, with variants called using GATK HaplotypeCaller (v4.0.12.0) with joint genotyping across all samples. Variant calls were restricted to SNPs on the 14 nuclear chromosomes, excluding indels and hypervariable regions, and stringent filtration was applied based on quality metrics (QD, MQ, MQRankSum, ReadPosRankSum, SOR), read depth, and missingness. Essentiality of genes for *in vitro* asexual blood stage development can be quantified using the piggyBac insertion mutagenesis score (MIS), which ranges from 0 for essential genes to 1 for dispensable genes[5]. To quantify false discovery rates for Nanopore and Illumina SNP calling, we restricted our attention to novel coding variants detected in the top quantile of essential genes (n=1356), with MIS in the range 0 to 0.142. Coding effects of each SNP were predicted using the R (V3.6.1) package *VariantAnnotation V1.32.*0[6].

Supplementary Text 5: Principal Coordinates Analysis (PCoA) of Isolates from PNG and South-East Asia

Nanopore-sequenced field isolates were genotyped at 742,365 high-quality SNP loci obtained through our in-house reanalysis of the MalariaGEN Pf3k/PNG dataset. Only Nanopore genotypes with depth of coverage at least 2X and a minimum of 75% of reads supporting the called genotype were retained. Single-sample VCF files for Nanopore-sequenced field isolates were merged into a multisample VCF file using *vcftools V0.1.13*[7]. Multisample VCF files for both the MalariaGEN Pf3k/PNG datasets and our Nanopore-sequenced field isolates were then converted into *gds* format[8] and imported into R statistical software (V3.6.2)[9].

Isolates with genotype missingness rates exceeding 30% were removed, yielding 10 MinION-sequenced field isolates (all originating from Papua New Guinea) and 1040 Illumina-sequenced field isolates. Variant-level filtration was then performed to retain only SNP loci with missingness <10%, leaving 298,112 variants. SNPs monomorphic across the filtered panel of field isolates were removed to yield 51,421 polymorphic high-quality SNPs. These filtration steps were performed using *SeqArray V1.22.3*[10].

Pairwise distances between the resulting genotypes, defined to be the proportion of differing SNP sites between each pair of isolates, were computed using the *dist-gene* function in the R package *ape V5.2*[11]; missing sites were excluded in pairwise comparisons. Principal coordinates analysis (PCoA) was performed on the resultant pairwise distance matrix with the base R function *cmdscale (*V3.6.2) with default parameters to examine clustering patterns stratified by geographic origin and sequencing platform.

To examine the relationship between Nanopore-sequenced field isolates and Illumina-sequenced isolates exclusively from Papua New Guinea, a separate PCoA was performed restricted to PNG samples using the same 51,421 polymorphic SNP loci. One Nanopore-sequenced PNG isolate identified as an outlier in the full PCoA was excluded, yielding 9 Nanopore-sequenced and 1,040 Illumina-sequenced field isolates. All subsequent distance computation and ordination steps were performed as described above. Isolates were stratified by sequencing platform (Nanopore or Illumina) and geographic origin for visualisation.

Supplementary Text 6: Benchmarking of BB12 *de novo* Assemblies Against the IT Reference Genome

Since the laboratory isolate BB12 is a descendent of the IT strain of *Plasmodium falciparum*, we expect the core nuclear genomes of BB12 and IT to exhibit a high degree of similarity. Our preliminary benchmark for *de novo* assembly was thus a contig-level alignment of a potential BB12 assembly to the IT4 reference genome (version 4)[12]. We sought to minimise indel and mismatch rates for draft BB12 assemblies compared (Figure S1B) to the IT4 reference genome, generating quality summaries for various pipeline stages using *BUSCO V.5*[13].

The initial scaffold, generated using *Flye V.2.9.3* from raw Nanopore reads, had high continuity, with all but one nuclear chromosome spanned by a single contig and two spurious contigs. Ten iterations of consensus polishing with *Racon V1.5.0*[15] and *Medaka V.1.11.3* substantially improved alignment (mismatch rates) against the IT reference genome. To correct small-scale errors and local misassemblies, we further polished this draft assembly with Illumina data using *Pilon V.1.22*[16]. The resultant hybrid Nanopore-Illumina assembly exhibited slightly decreased indel error (a characteristics of a short-read assembly) compared to Nanopore only assembly but had comparable mismatch rates, with 191 indels and 14 mismatches per 100kb (Supplementary Table 5).

Supplementary Text 7: Gene Annotation and *var* Gene Domain Classification
Gene annotation of hybrid assemblies was performed with the *Companion* pipeline[17] via the web server (https://companion.sanger.ac.uk) (July 2024 version), using *P. falciparum 3D7* as a reference strain and taking into account reference protein evidence. Highly conserved genes were mapped using the Rapid Annotation Transfer Tool (RATT), while a relatively lenient threshold inclusion score of 0.5 was used for de novo gene prediction by AUGUSTUS, to increase the sensitivity of gene annotation. Pseudogene detection was also performed as part of the Companion pipeline.

To characterise the ability of Nanopore long reads to access complex genomic regions, genes encoding either PfEMP1, DBL-domains, N-terminal or acidic-terminal segments (all associated with *var* genes) were identified. Protein sequences of all potential full and partial *var* genes were extracted and classified using the *VarDom 1.0 Server*[18] (https://services.healthtech.dtu.dk/services/VarDom-1.0/), with the default lower-bit score threshold 9.97 for homology blocks. Annotated *var* genes were complete if they included both acidic- and N-terminal segments. Accounting for the possible disruption of ORFs due to local assembly errors, neighbouring annotated *var* candidates within 1200bp windows were concatenated if their domain structures suggested they corresponded to the same gene to obtain 'split' *var* genes. *Var* candidates that included at least 3 annotated domains but did not contain either acidic- or N-terminal segments were also considered to be partial *var* genes.

Supplementary Text 8: Structural Variant Calling Pipelines
For short read data (Illumina), adapter trimming was first performed with *TrimGalore V0.4.4*[19]. Trimmed Illumina reads were aligned against the 3D7 reference genome with *bwa mem V0.7.13*[3], and resultant alignments files were sorted and indexed with *Samtools V1.7*[20] utilities. Structural variant calling was then performed with *GRIDSS V2.8.3*[21]. Unpaired breakend variants were removed, and only simple variant types (i.e. insertions, deletions, duplications and inversions) were annotated using the R package *StructuralVariantAnnotation V1.0.0.* Structural variants were retained if they passed default quality filters (i.e. the FILTER flag in the VCF files generated by GRIDSS was set to PASS).

For long read data (Nanopore), reads were first aligned against the 3D7 reference genome using *minimap2*. The resultant alignments were sorted and indexed using *Samtools V1.7*[20] utilities. Structural variant calling was subsequently performed with *Sniffles V1*[22] using default parameters. Breakends were removed to retain only insertions, deletions, translocations, duplications and inversions. A threshold of 20 high-quality reads supporting the called variant (as determined by the RE tag in the output VCF file) was implemented to obtain high-quality structural variants. Overlapping duplications and deletions were assumed to be spurious and were accordingly removed.

To perform structural variant calling with our *de novo* assemblies, we first aligned our assemblies against 3D7 reference genome using *nucmer* (*MUMmer, V.3.1*[23]). All anchor matches were used, irrespective of their uniqueness, and a minimum cluster length of 500 was implemented; maximal exact matches were also required to be at least 100bp in length. The resultant delta files were processed using the Assemblytics command line version available at https://github.com/MariaNattestad/Assemblytics to identify structural variants.

To quantify concordance rates across these methodologies we compared the set of high-quality SVs detected by each pipeline using a custom *R* script. SVs identified by different pipelines were equivalent if: (i) they overlapped by at least 1bp; (ii) were annotated within the same SV class (Supplementary Table 5); and (iii) if the length of the shorter SV was at least 75% of the length of the longer SV. To account for systematic indel errors in MinION basecalling, particularly in homopolymeric tracts, we retained only SVs with length at least 200 bp[22]. SVs exceeding 300 kbp seemed to be spurious across all three pipelines and were thus removed.

REFERENCES

1. Van der Auwera GA*, et al.* From FastQ data to high confidence variant calls: the Genome Analysis Toolkit best practices pipeline. *Curr Protoc Bioinformatics* **43**, 11.10.11-11.10.33 (2013).

2. Institute B. Picard Tools. (ed^(eds). GitHub.

3. Li H. Aligning sequence reads, clone sequences and assembly contigs with BWA-MEM. *arXiv preprint arXiv:13033997*, (2013).

4. McKenna A*, et al.* The Genome Analysis Toolkit: a MapReduce framework for analyzing next-generation DNA sequencing data. *Genome Res* **20**, 1297-1303 (2010).

5. Zhang M*, et al.* Uncovering the essential genes of the human malaria parasite. *Science* **360**, (2018).

6. Obenchain V, Lawrence M, Carey V, Gogarten S, Shannon P, Morgan M. VariantAnnotation: a Bioconductor package for exploration and annotation of genetic variants. *Bioinformatics* **30**, 2076-2078 (2014).

7. Danecek P*, et al.* The variant call format and VCFtools. *Bioinformatics* **27**, 2156-2158 (2011).

8. Zheng X, Levine D, Shen J, Gogarten SM, Laurie C, Weir BS. A high-performance computing toolset for relatedness and principal component analysis of SNP data. *Bioinformatics* **28**, 3326-3328 (2012).

9. Team RC. R: A language and environment for statistical computing. (ed^(eds). R Foundation for Statistical Computing (2013).

10. Zheng X*, et al.* SeqArray-a storage-efficient high-performance data format for WGS variant calls. *Bioinformatics* **33**, 2251-2257 (2017).

11. Paradis E, Schliep K. ape 5.0: an environment for modern phylogenetics and evolutionary analyses in R. *Bioinformatics* **35**, 526-528 (2019).

12. Otto TD*, et al.* Long read assemblies of geographically dispersed. *Wellcome Open Res* **3**, 52 (2018).

13. Gurevich A, Saveliev V, Vyahhi N, Tesler G. QUAST: quality assessment tool for genome assemblies. *Bioinformatics* **29**, 1072-1075 (2013).

14. Koren S, Walenz BP, Berlin K, Miller JR, Bergman NH, Phillippy AM. Canu: scalable and accurate long-read assembly via adaptive. *Genome Res* **27**, 722-736 (2017).

15. Vaser R, Sović I, Nagarajan N, Šikić M. Fast and accurate de novo genome assembly from long uncorrected reads. *Genome Res* **27**, 737-746 (2017).

16. Walker BJ*, et al.* Pilon: an integrated tool for comprehensive microbial variant detection and genome assembly improvement. *PLoS One* **9**, e112963 (2014).

17. Steinbiss S*, et al.* Companion: a web server for annotation and analysis of parasite genomes. *Nucleic Acids Res* **44**, W29-34 (2016).

18. Rask TS, Hansen DA, Theander TG, Gorm Pedersen A, Lavstsen T. Plasmodium falciparum erythrocyte membrane protein 1 diversity in seven genomes--divide and conquer. *PLoS Comput Biol* **6**, (2010).

19. Krueger F. TrimGalore. (ed^(eds). GitHub.

20. Li H*, et al.* The Sequence Alignment/Map format and SAMtools. *Bioinformatics* **25**, 2078-2079 (2009).

21. Cameron DL*, et al.* GRIDSS: sensitive and specific genomic rearrangement detection using positional de Bruijn graph assembly. *Genome Res* **27**, 2050-2060 (2017).

22. Sedlazeck FJ*, et al.* Accurate detection of complex structural variations using single-molecule sequencing. *Nat Methods* **15**, 461-468 (2018).

23. Kurtz S*, et al.* Versatile and open software for comparing large genomes. *Genome Biol* **5**, R12 (2004).

24. Nattestad M, Schatz MC. Assemblytics: a web analytics tool for the detection of variants from an assembly. *Bioinformatics* **32**, 3021-3023 (2016).

25. Tessema SK, et al. Antibodies to Intercellular Adhesion Molecule 1-Binding Plasmodium falciparum Erythrocyte Membrane Protein 1-DBLβ Are Biomarkers of Protective Immunity to Malaria in a Cohort of Young Children from Papua New Guinea. Infect Immun 86, (2018).

26. MalariaGEN, Ahouidi A, Ali M, et al. An open dataset of *Plasmodium falciparum* genome variation in 7,000 worldwide samples. *Wellcome Open Res* 6, 42 (2021).

27. Gardner MJ, et al. Genome sequence of the human malaria parasite *Plasmodium falciparum*. *Nature* 419, 498–511 (2002).

SUPPLEMENTARY FIGURES

Supplementary Figure 1. Experimental workflow and optimised bioinformatic pipelines
Supplementary Figure 2. Cumulative coverage curves for *P. falciparum* laboratory isolates and mock infection samples sequenced on the Nanopore MinION platform.
Supplementary Figure 3. Receiver operating characteristic (ROC) curve showing the predictive performance of *Plasmodium falciparum* parasite copy number for Nanopore sequencing success
Supplementary Figure 4. Parameters associated with Nanopore sequencing output, by flow cell
Supplementary Figure 5. Relative frequencies of concordant and discordant genotype calls using Nanopore and Illumina data
Supplementary Figure 6. Alignments of de novo BB12 and XHA assemblies against reference genomes
Supplementary Figure 7. Domain structures for annotated *var* genes

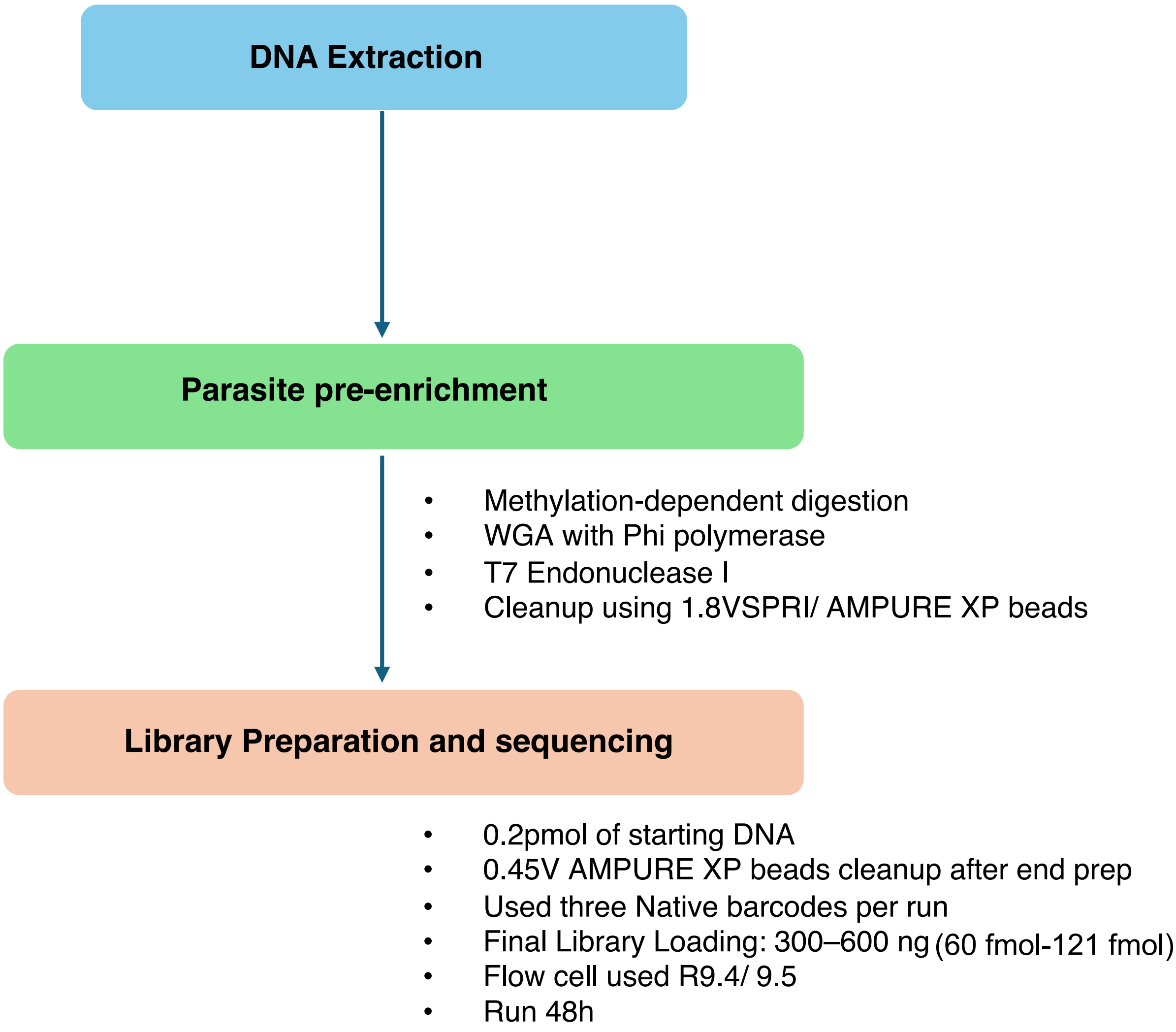

Supplementary Figure 1A. Experimental workflow of MinION sequencing for *Plasmodium falciparum* isolates

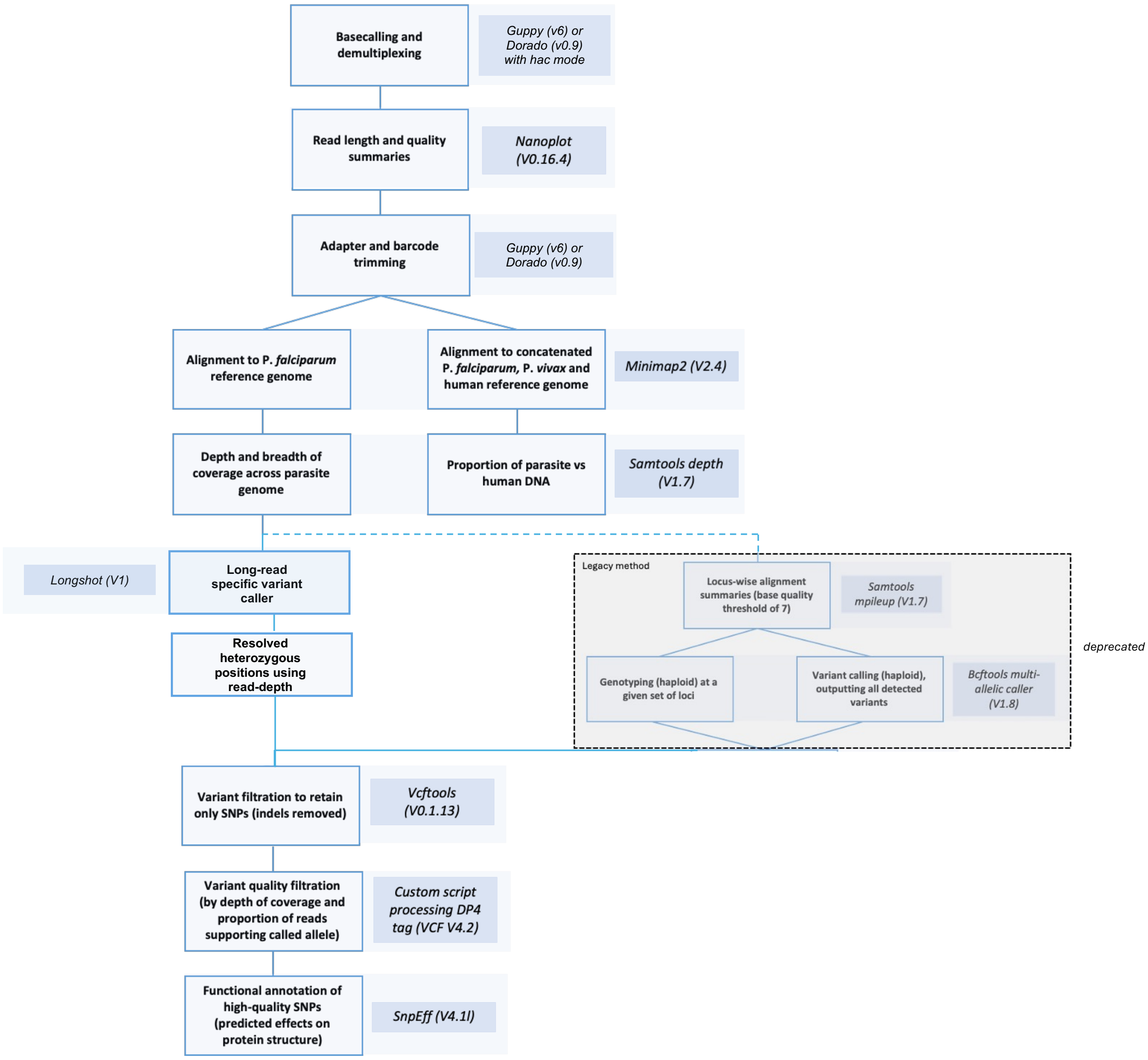

Supplementary Figure 1B. Variant calling and alignment pipeline for Nanopore data.

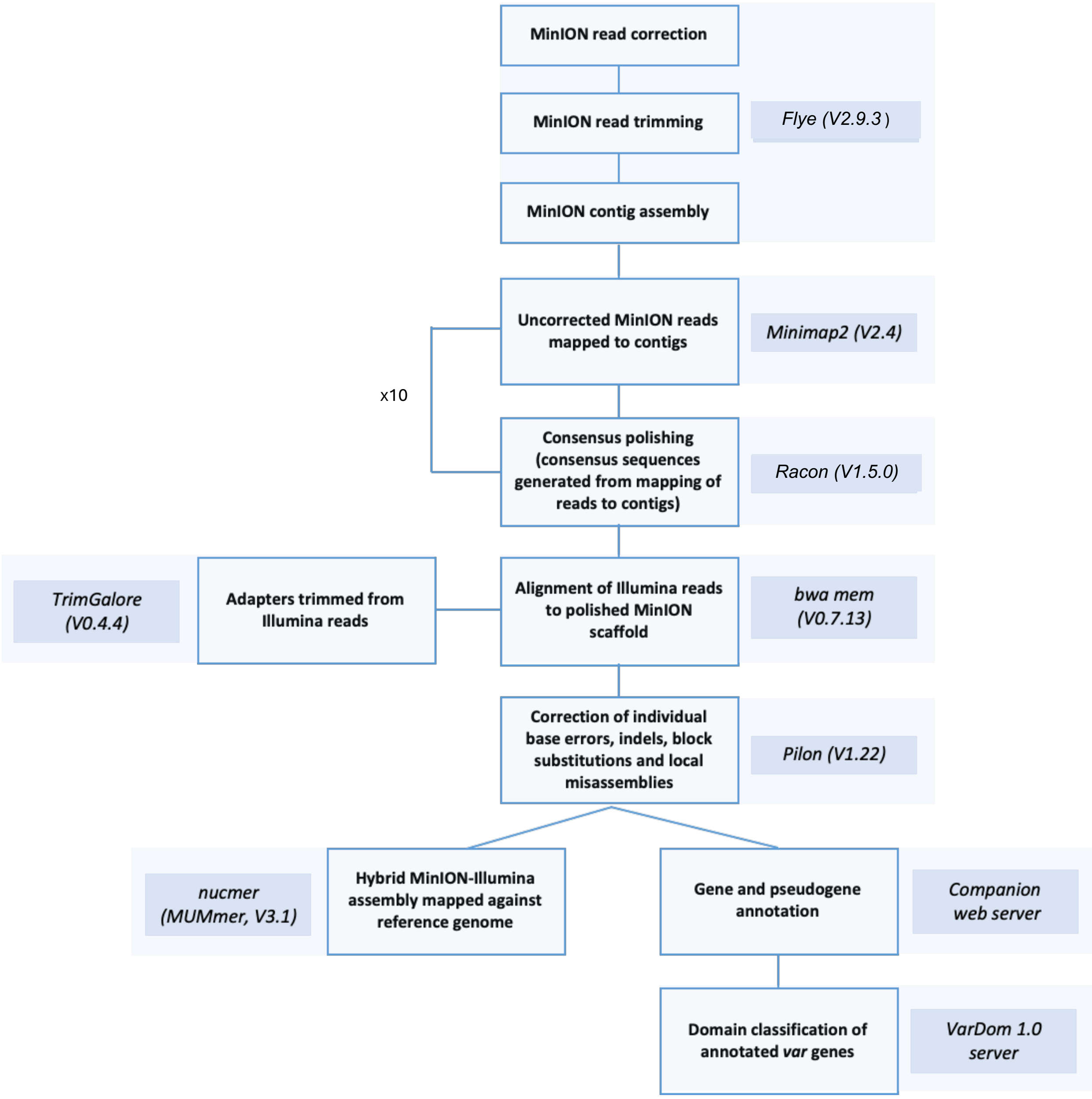

Supplementary Figure 1C. *De novo* assembly pipeline for hybrid assemblies of Nanopore and Illumina data

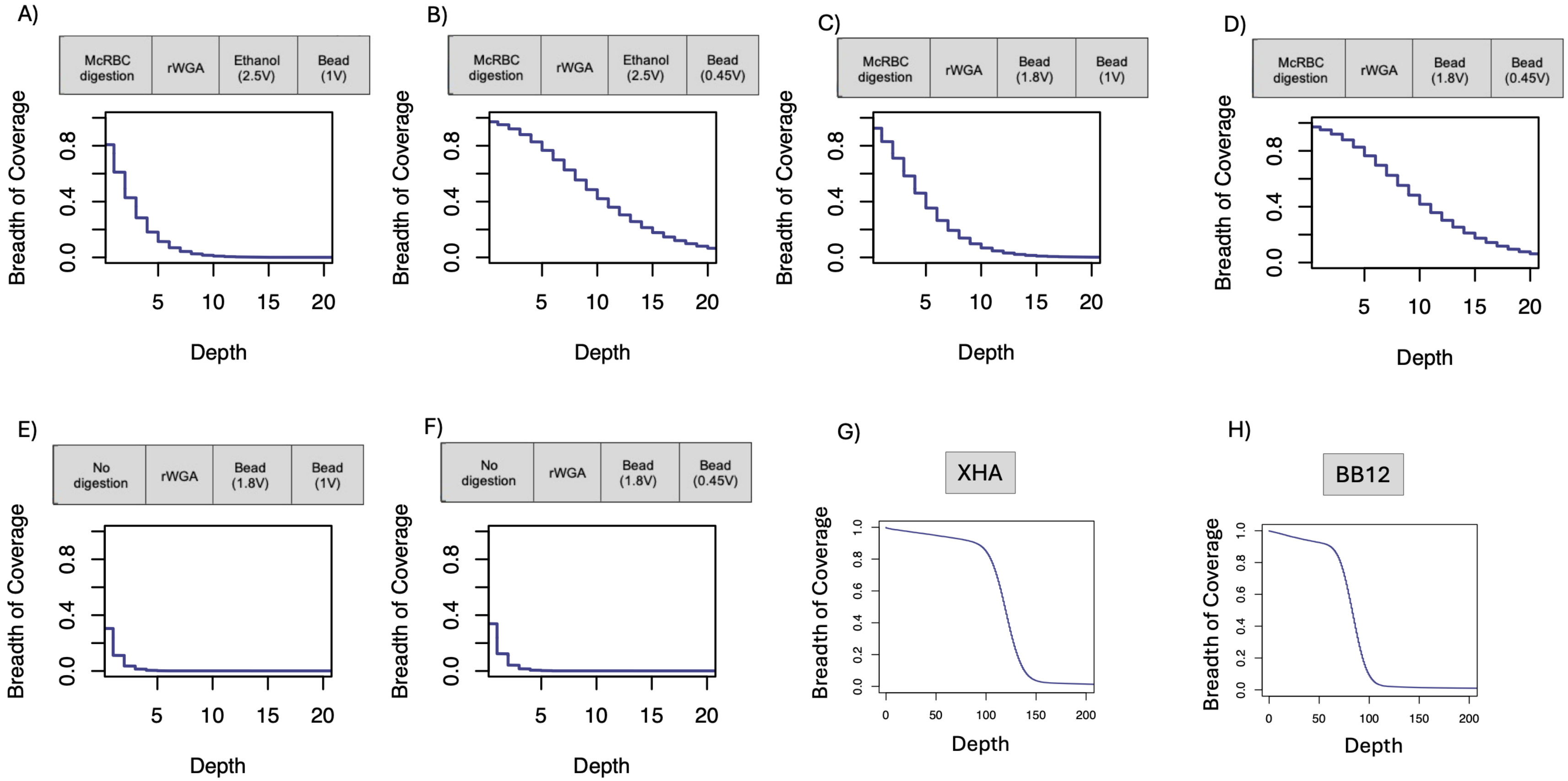

Supplementary Figure 2. Cumulative coverage curves for *P. falciparum* laboratory isolates and mock infections samples sequenced on the Nanopore MinION platform. Panels show cumulative distribution plots of the proportion of the parasite reference genome covered at increasing sequencing depths (breadth of coverage vs. depth), providing an assessment of evenness of sequencing coverage. (A–F) Coverage curves for six mock infection conditions, each comprising 1% *P. falciparum* (3D7) DNA spiked into human DNA and subject to random whole-genome amplification (rWGA) with varying enrichment and purification strategies (A) McrBC digestion prior to rWGA with ethanol (2.5V) and bead (1V) cleanup; (B) McrBC digestion with ethanol (2.5V) and bead (0.45V) cleanup after end-prep; (C) McrBC digestion with bead (1.8V) and bead (1V) cleanup after end-prep; (D) McrBC digestion with bead (1.8V) and bead (0.45V) cleanup after end-prep; (E) no digestion with bead (1.8V) and bead (1V) cleanup after end-prep; and (F) no digestion with bead (1.8V) and bead (0.45V) cleanup after end-prep. V denotes the ratio of bead or ethanol volume to sample volume. All six conditions were sequenced in multiplex. Coverage curves for pure *P. falciparum* laboratory isolates XHA (G) and BB12 (H), demonstrating high and uniform breadth of coverage maintained across sequencing depths up to 200×.

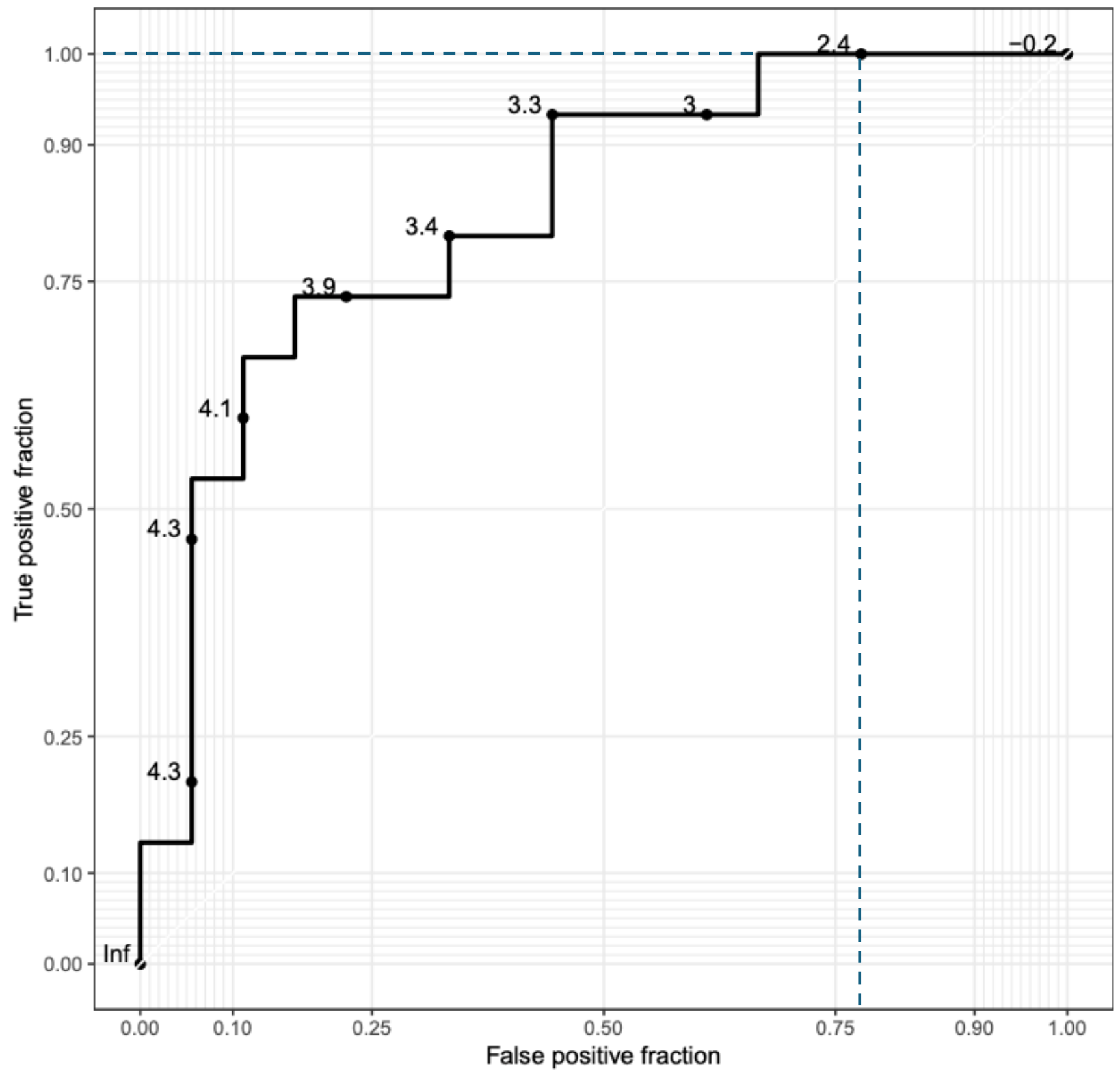

Supplementary Figure 3. Receiver operating characteristic (ROC) curve showing the predictive performance of *Plasmodium falciparum* parasite copy number for Nanopore sequencing success. The binary outcome variable was defined as samples achieving ≥75% genome coverage at 1× depth. True positive indicates samples with a given parasite density that achieved the minimum coverage, and false positive indicates samples that did not. Parasite densities were log_{10}-transformed prior to analysis, and each point on the curve represents individual samples, plotted as log_{10}(copies/μL). Higher log_{10} values correspond to higher parasite densities, whereas lower values reflect lower parasite loads. The dotted vertical line indicates the optimal threshold (log_{10} 2.4 ≈ 2.5 × 10^2 copies/μL), which provided the highest combined sensitivity and specificity for predicting sequencing success at ≥75% genome coverage at 1× depth. This cutoff achieved an area under the curve (AUC) of 0.92 (95% CI, 0.81–1.00).

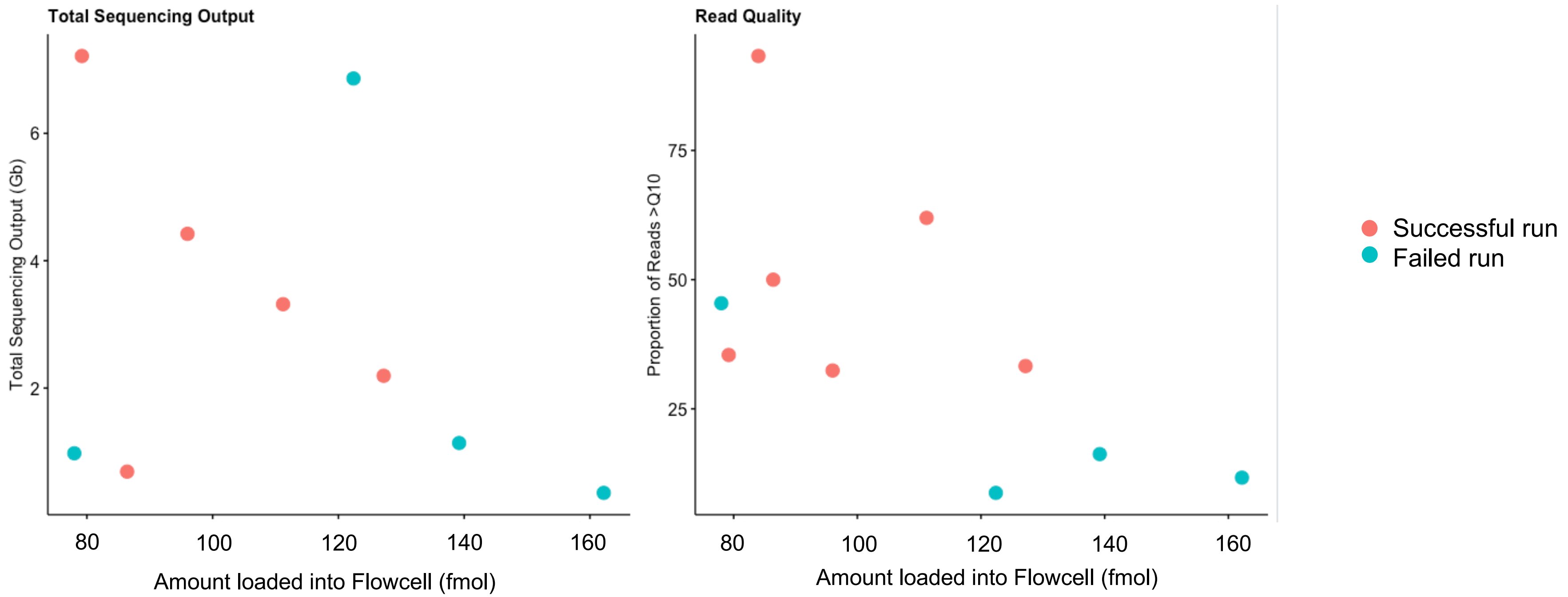

Supplementary Figure 4. Parameters associated with Nanopore sequencing output, by flowcell. Nanopores tend to become saturated when large amounts of DNA (femtomoles) are loaded into a flowcell, leading to a generally decreasing trend between the total sequencing output and quality and the molarity of loaded DNA. Runs were classified as failed if total sequencing output was less than 2 GB and the proportion of reads with quality score > Q10 was less than 50%. The colours indicate whether the run failed (blue) or was successful (red) as indicated in the key.

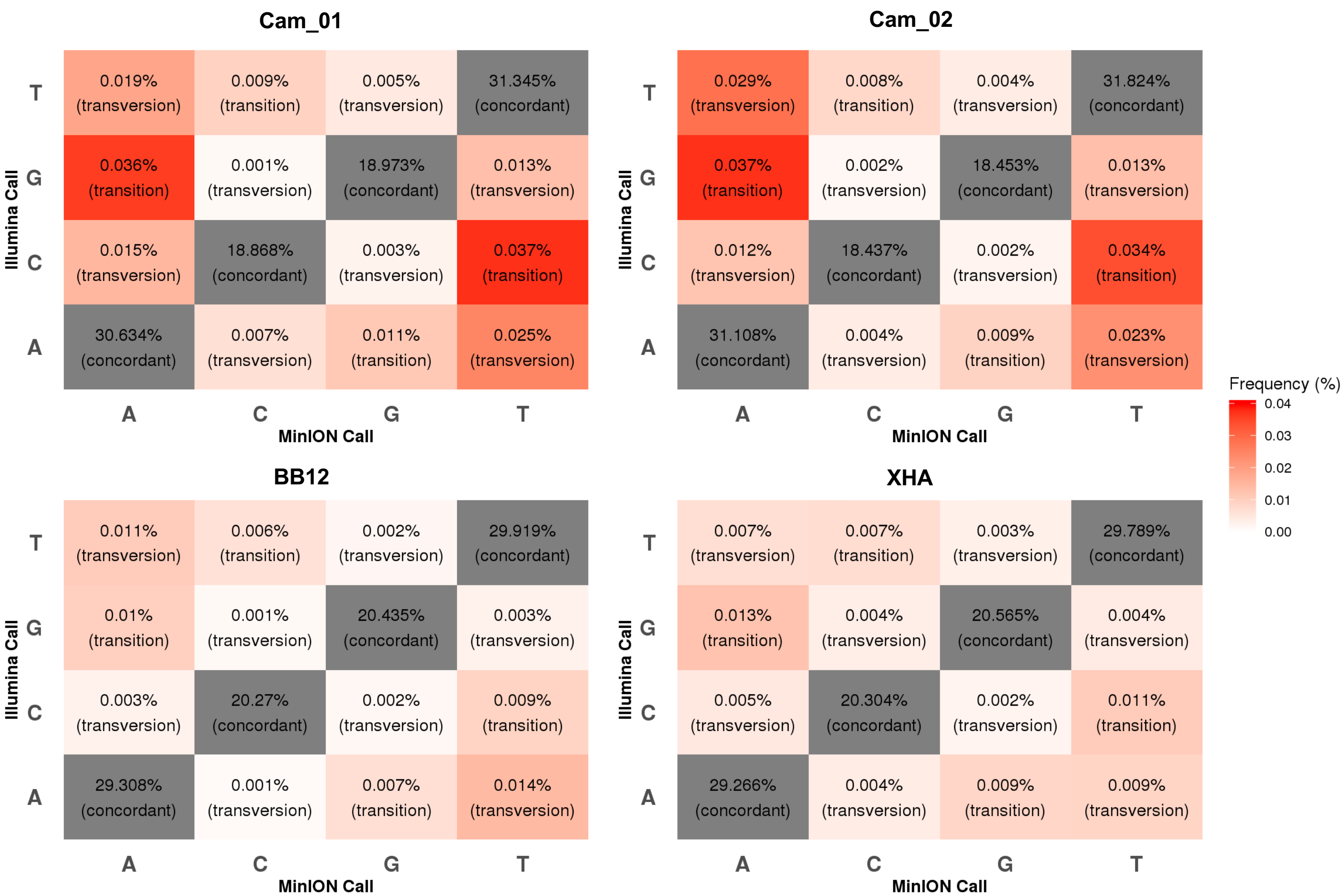

Supplementary Figure 5. Relative frequencies of concordant and discordant genotype calls using Nanopore and Illumina data. Discordant calls were stratified by transition/transversion. Transition errors (between bases with similar ring structures) tend to be more common than transversion errors.

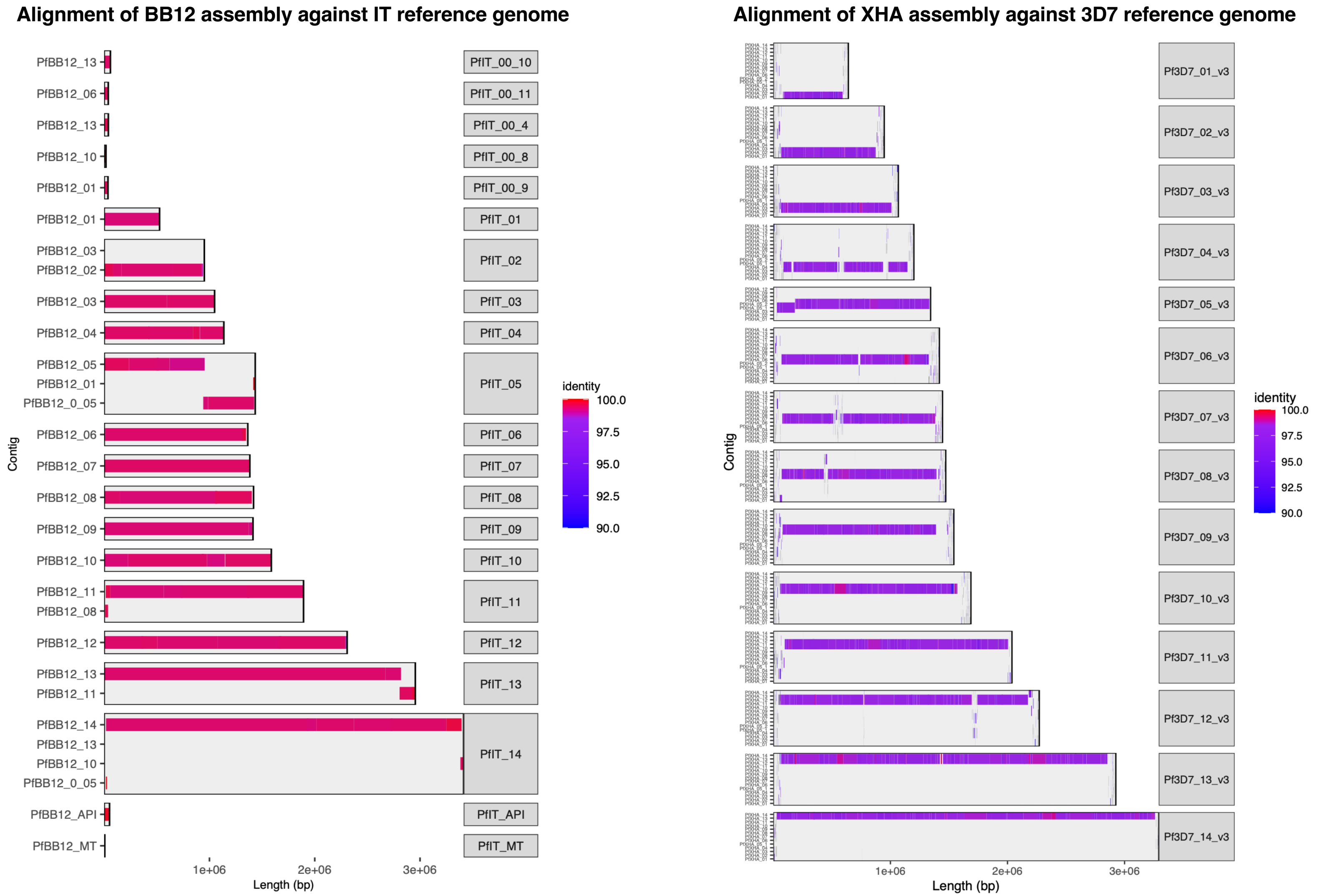

Supplementary Figure 6. Alignments of *de novo* BB12 and XHA assemblies against reference genomes

BB12 and XHA de novo assemblies were aligned to IT4 (version 4) and 3D7 (version 3) respectively, with one-to-one mappings between de novo contigs and reference chromosomes computed using nucmer (MUMmer, V.3.1). Assembly continuity is high, with all but one nuclear chromosome spanned by a single contig. De novo contigs and reference genomes are generally concordant in core genomic regions, but alignments tend to become fragmented in subtelomeric hypervariable regions.

A.

COMPLETE (n=27)														
Contig	Dir	Start	End	Gene(s)	Domain Structure									
tig00000031	+	387210	398129	Pf_050041100	NTS	DBLa	CIDRa	DBLb	DBLg	DBLe	DBLg	DBLz	DBLe	ATS
tig00000027	-	1014328	1024713	Pf_030030800	NTS	DBLa	CIDRa	DBLd	CIDRg	DBLe	DBLz	DBLe	ATS	
tig00000020	-	38859	49502	Pf_070006100	NTS	DBLa	CIDRg	DBLb	DBLd	CIDRg	DBLz	DBLe	ATS	
tig00000004	-	48848	58897	Pf_120006700	NTSpam	DBLpam1	DBLpam2	CIDRpam	DBLpam3	DBLe	DBLe	DBLe	ATS	
tig00000039	-	3319224	3331125	Pf_140087500	NTS	DBLa	CIDRa	DBLb	DBLd	CIDRg	DBLg	DBLz	ATS	
tig00000029	+	19757	29476	Pf_020008600	NTS	DBLa	CIDRa	DBLb	DBLd	CIDRg	DBLg	ATS		
tig00000004	+	2254349	2263040	Pf_120062500	NTS	DBLa	CIDRb	DBLg	DBLz	DBLe	DBLe	ATS		
tig00000019	-	702626	711217	Pf_060022400	NTS	DBLa	CIDRa	DBLb	DBLg	DBLd	CIDRg	ATS		
tig00000023	+	29825	38421	Pf_050006200	NTS	DBLa	CIDRa	DBLb	DBLd	CIDRb	ATS			
tig00000004	+	1723341	1731362	Pf_120047200	NTS	DBLa	CIDRa	DBLe	DBLz	DBLe	ATS			
tig00000006	-	1976855	1985905	Pf_110054400	NTS	DBLa	CIDRa	DBLb	DBLd	CIDRb	ATS			
tig00000002	-	31911	41304	Pf_130006000	NTS	DBLa	DBLg	DBLe	DBLd	CIDRb	ATS			
tig00000027	+	20928	28104	Pf_030005200	NTS	DBLa	CIDRa	DBLd	CIDRb	ATS				
tig00000016	+	397087	404890	Pf_080013800	NTS	DBLa	CIDRa	DBLd	CIDRb	ATS				
tig00000006	+	22738	30018	Pf_110005700	NTS	DBLa	CIDRa	DBLd	CIDRb	ATS				
tig00000004	+	26055	34261	Pf_120006200	NTS	DBLa	CIDRa	DBLd	CIDRb	ATS				
tig00000004	+	1695282	1702784	Pf_120046700	NTS	DBLa	CIDRa	DBLd	CIDRb	ATS				
tig00000004	+	1714428	1721923	Pf_120047100	NTS	DBLa	CIDRa	DBLd	CIDRb	ATS				
tig00000025	-	873055	879737	Pf_040028600	NTS	DBLa	CIDRa	DBLd	CIDRb	ATS				
tig00000025	-	912273	920409	Pf_040029100	NTS	DBLa	CIDRa	DBLd	CIDRb	ATS				
tig00000025	-	1106037	1113680	Pf_040034300	NTS	DBLa	CIDRa	DBLd	CIDRg	ATS				
tig00000031	-	419356	426549	Pf_050041900	NTS	DBLa	CIDRa	DBLd	CIDRb	ATS				
tig00000004	-	776658	784284	Pf_120025500	NTS	DBLa	CIDRa	DBLd	CIDRb	ATS				
tig00000002	-	2856518	2863633	Pf_130078700	NTS	DBLa	CIDRa	DBLd	CIDRb	ATS				
tig00000033	-	560062	567221	Pf_010019500	NTS	DBLa	CIDRa	DBLb	ATS					
tig00000025	-	884420	889761	Pf_040028700	NTS	DBLa	CIDRa	ATS						
tig00000039	-	61109	65241	Pf_140006500	NTS	DBLa	DBLe	ATS						

SPLIT (n=16)														
Contig	Dir	Start	End	Gene(s)	Domain Structure									
tig00000027	+	1002348	1013225	Pf_030030600,Pf_030030700	NTS	DBLa	CIDRa	DBLb	DBLg	DBLg	DBLd	CIDRb	791bp	ATS
tig00000019	+	1345115	1356162	Pf_060038200,Pf_060038300	NTS	DBLa	CIDRa	DBLb	DBLb	DBLg	DBLd	CIDRb	704bp	ATS
tig00000042	+	1539652	1550184	Pf_100044600,Pf_100044700	NTS	DBLa	CIDRa	DBLb	DBLg	DBLg	DBLd	CIDRb	675bp	ATS
tig00000020	-	506384	517153	Pf_070017300,Pf_070017200	NTS	DBLa	CIDRa	DBLb	DBLd	CIDRg	DBLg	DBLz	651bp	ATS
tig00000042	-	1551316	1561180	Pf_100044900,Pf_100044800	NTS	DBLa	CIDRa	DBLb	DBLg	DBLd	CIDRb	981bp	ATS	
tig00000020	-	548018	557889	Pf_070018000,Pf_070017900	NTS	DBLa	CIDRa	25bp	DBLg	DBLd	CIDRb	ATS		
tig00000004	-	765441	775082	Pf_120025400,Pf_120025300	NTS	DBLa	CIDRa	DBLb	83bp	DBLd	CIDRb	ATS		
tig00000020	+	28789	37802	Pf_070005900,Pf_070006000	NTS	145bp	CIDRa	DBLb	DBLd	CIDRb	ATS			
tig00000004	+	1757379	1764810	Pf_120048000,Pf_120048100	NTS	42bp	DBLa	CIDRa	DBLd	CIDRb	ATS			
tig00000025	-	533267	540934	Pf_040020600,Pf_040020500	NTS	42bp	DBLa	CIDRa	DBLd	CIDRb	ATS			
tig00000025	-	896785	904808	Pf_040028900,Pf_040028800	NTS	DBLa	CIDRa	DBLd	CIDRb	212bp	ATS			
tig00000019	-	1357247	1366157	Pf_060038600,Pf_060038400	NTS	885bp	CIDRa	DBLg	DBLd	CIDRb	ATS			
tig00000020	-	537548	546613	Pf_070017800,Pf_070017700	NTS	DBLa	CIDRa	DBLb	56bp	CIDRb	ATS			
tig00000004	-	2264049	2271729	Pf_120062700,Pf_120062600	NTS	DBLa	CIDRa	DBLd	CIDRb	101bp	ATS			
tig00000016	+	412725	420025	Pf_080014000,Pf_080014100	NTS	216bp	CIDRa	DBLd	CIDRb	ATS				
tig00000004	+	1737785	1745602	Pf_120047400,Pf_120047500,Pf_120047700	NTS	DBLa	94bp	DBLd	480bp	ATS				

PARTIAL (n=11)														
Contig	Dir	Start	End	Gene(s)	Domain Structure									
tig00000002	+	20623	30908	Pf_130005900	–	DBLa	CIDRa	DBLb	DBLd	CIDRg	DBLg	DBLz	ATS	
tig00000020	-	519887	528754	Pf_070017500,Pf_070017400	–	CIDRa	DBLb	DBLg	DBLd	CIDRb	952bp			
tig00000020	+	559875	568672	Pf_070018100	–	DBLa	CIDRd	DBLb	DBLg	DBLg	ATS			
tig00000025	+	29805	34781	Pf_040008200	NTS	DBLa	CIDRa	DBLd	CIDRb	–				
tig00000039	+	39615	44859	Pf_140006200	NTS	DBLa	CIDRa	DBLd	CIDRg	–				
tig00000033	-	573756	576278	Pf_010020100,Pf_010020000,Pf_010019900	NTS	26bp	DBLa	125bp	CIDRa	–				
tig00000025	-	508033	513473	Pf_040020200	NTS	DBLa	CIDRa	DBLd	CIDRg	–				
tig00000025	-	523080	528397	Pf_040020400	NTS	DBLa	CIDRa	DBLd	CIDRb	–				
tig00000020	-	572106	577367	Pf_070018200	NTS	DBLa	CIDRa	DBLd	CIDRb	–				
tig00000016	-	1356800	1361793	Pf_080038100	NTS	DBLa	CIDRa	DBLd	CIDRb	–				
tig00000029	-	904465	907349	Pf_020031500	NTS	DBLa	CIDRa	–						

B.

COMPLETE (n=18)														
Contig	Dir	Start	End	Gene(s)	Domain Structure									
tig00000013	-	546257	557851	PfXHA_070018300	NTS	DBLa	CIDRa	DBLb	DBLg	DBLd	CIDRb	DBLb	DBLg	ATS
tig00000021	+	1030844	1041909	PfXHA_030031500	NTS	DBLa	CIDRa	DBLb	DBLb	DBLg	DBLd	CIDRb	ATS	
tig00000013	+	37217	48841	PfXHA_070006100	NTS	DBLa	CIDRa	DBLb	DBLd	DBLe	DBLz	DBLe	ATS	
tig00000011	+	1558431	1568495	PfXHA_100045800	NTS	DBLa	CIDRg	DBLg	DBLe	DBLe	DBLz	DBLe	ATS	
tig00000018	+	30296	39580	PfXHA_040006000	NTS	DBLa	CIDRa	DBLb	DBLg	DBLe	DBLe	ATS		
tig00000022	-	40802	49789	PfXHA_020006500	NTS	DBLa	CIDRg	DBLg	DBLe	DBLz	DBLe	ATS		
tig00000013	-	535465	544837	PfXHA_070018200	NTS	DBLa	CIDRa	DBLb	DBLg	DBLz	DBLe	ATS		
tig00000006	-	31586	40888	PfXHA_110006200	NTS	DBLa	DBLb	DBLg	DBLz	DBLe	ATS			
tig00000022	+	32205	39752	PfXHA_020006400	NTS	DBLa	CIDRa	DBLd	CIDRb	ATS				
tig00000026	+	30809	39061	PfXHA_050005800	NTS	DBLa	CIDRa	DBLz	DBLe	ATS				
tig00000004	+	20145	27651	PfXHA_120005600	NTS	DBLa	CIDRa	DBLd	CIDRb	ATS				
tig00000004	+	1702252	1709913	PfXHA_120047000	NTS	DBLa	CIDRa	DBLd	CIDRb	ATS				
tig00000018	-	529655	537112	PfXHA_040018500	NTS	DBLa	CIDRa	DBLd	CIDRb	ATS				
tig00000018	-	543735	550646	PfXHA_040018800	NTS	DBLa	CIDRa	DBLd	CIDRb	ATS				
tig00000018	-	887073	893998	PfXHA_040026900	NTS	DBLa	CIDRa	DBLd	CIDRb	ATS				
tig00000018	-	900996	908480	PfXHA_040027000	NTS	DBLa	CIDRa	DBLd	CIDRb	ATS				
tig00000011	-	1569528	1576839	PfXHA_100045900	NTS	DBLa	CIDRa	DBLd	CIDRb	ATS				
tig00000007	-	40449	44425	PfXHA_090005900	NTS	DBLa	DBLe	ATS						

SPLIT (n=16)														
Contig	Dir	Start	End	Gene(s)	Domain Structure									
tig00000006	-	2013307	2023745	PfXHA_110056400,PfXHA_110056300	NTS	DBLa	CIDRa	DBLd	CIDRg	DBLz	DBLe	47bp	DBLe	ATS
tig00000003	+	2865002	2875282	fXHA_130082400,PfXHA_130082500,PfXHA_13008260	NTSpam	DBLpam1	DBLpam2	86bp	DBLpam3	DBLe	41bp	DBLe	ATS	
tig00000004	-	755457	765304	fXHA_120025600,PfXHA_120025500,PfXHA_12002540	NTS	DBLa	CIDRa	78bp	CIDRg	DBLz	DBLe	728bp	ATS	
tig00000009	+	438011	445181	fXHA_080014400,PfXHA_080014500,PfXHA_08001460	NTS	DBLa	CIDRa	53bp	DBLd	CIDRb	974bp	ATS		
tig00000018	-	509413	518281	PfXHA_040018200,PfXHA_040018100	NTS	DBLa	CIDRa	DBLd	48bp	CIDRg	DBLg	ATS		
tig00000018	-	933994	941397	fXHA_040027700,PfXHA_040027600,PfXHA_04002750	NTS	DBLa	47bp	CIDRa	104bp	DBLd	CIDRb	ATS		
tig00000013	-	49883	57659	fXHA_070006400,PfXHA_070006300,PfXHA_07000620	NTS	DBLa	CIDRd	119bp	DBLz	82bp	DBLe	ATS		
tig00000014	+	31216	38915	fXHA_060006000,PfXHA_060006100,PfXHA_06000620	NTS	DBLa	CIDRa	113bp	CIDRb	35bp	ATS			
tig00000009	+	446609	453754	PfXHA_080014700,PfXHA_080014800	NTS	DBLa	CIDRa	DBLb	DBLg	27bp	ATS			
tig00000004	+	1760468	1768339	PfXHA_120048000,PfXHA_120048100	NTS	DBLa	CIDRa	DBLd	CIDRb	965bp	ATS			
tig00000022	-	894075	901932	PfXHA_020028800,PfXHA_020028700	NTS	DBLa	CIDRa	41bp	DBLd	CIDRb	ATS			
tig00000018	-	909903	917526	PfXHA_040027200,PfXHA_040027100	NTS	DBLa	CIDRa	DBLd	CIDRb	1099bp	ATS			
tig00000009	+	461595	469409	PfXHA_080015000,PfXHA_080015100	NTS	DBLa	CIDRa	38bp	CIDRb	ATS				
tig00000024	-	624400	632131	PfXHA_010022700,PfXHA_010022500	NTS	DBLa	823bp	DBLd	CIDRb	ATS				
tig00000013	-	503845	511830	PfXHA_070017500,PfXHA_070017400	NTS	DBLa	78bp	DBLd	CIDRb	ATS				
tig00000013	-	519595	526993	PfXHA_070017900,PfXHA_070017700	NTS	DBLa	CIDRa	DBLd	794bp	ATS				

PARTIAL (n=15)														
Contig	Dir	Start	End	Gene(s)	Domain Structure									
tig00000004	-	35603	45661	PfXHA_120006000,PfXHA_120005900	–	DBLpam1	DBLpam2	CIDRpam	DBLpam3	DBLe	DBLe	DBLe	449bp	ATS
tig00000004	+	2227867	2235876	PfXHA_120061600	NTSpam	DBLpam1	DBLpam2	CIDRpam	DBLpam3	DBLe	DBLe	DBLe	–	
tig00000014	-	40698	51096	PfXHA_060006400,PfXHA_060006300	NTS	DBLa	CIDRa	DBLb	DBLg	121bp	DBLd	CIDRb	–	
tig00000009	-	1428724	1436962	PfXHA_080040100	NTS	DBLa	CIDRa	DBLb	DBLg	DBLd	CIDRb	DBLb	–	
tig00000003	-	27257	35470	PfXHA_130005700	NTS	DBLa	CIDRa	DBLb	DBLg	DBLd	CIDRb	DBLb		
tig00000003	+	37206	45155	PfXHA_130005800,PfXHA_130005900	–	DBLb	DBLb	DBLd	CIDRb	923bp	ATS	–		
tig00000024	+	45147	50224	PfXHA_010006700	NTS	DBLa	CIDRa	DBLd	CIDRb	–				
tig00000004	+	1683153	1688338	PfXHA_120046600	NTS	DBLa	CIDRa	DBLd	CIDRg	–				
tig00000018	-	927694	932618	PfXHA_040027400	NTS	DBLa	CIDRa	DBLd	CIDRb	–				
tig00000003	-	2882350	2887594	PfXHA_130082800	NTS	DBLa	CIDRa	DBLd	CIDRb	–				
tig00000006	+	25165	30487	PfXHA_110006100	–	DBLd	CIDRb	ATS						
tig00000001	+	29641	34407	PfXHA_140005900	–	DBLd	CIDRb	ATS						
tig00000014	-	745910	748894	PfXHA_060024500	NTS	DBLa	CIDRa	–						
tig00000013	-	564692	566983	PfXHA_070018700	NTS	DBLa	CIDRa	–						
tig00000004	-	2250204	2256010	PfXHA_120062100	–	DBLd	CIDRg	ATS						

Supplementary Figure 7. Domain structures for annotated *var* genes

Var gene structures were identified after *de novo* assembly of reads for *P. falciparum* laboratory isolates A) BB12 B) XHA.

SUPPLEMENTARY TABLES

Supplementary Table 1. Summary of flow cells and library preparation kits
Supplementary Table 2. Results of sequencing experiments
Supplementary Table 3. SNV genotyping concordance rates for two laboratory and two field isolates in homopolymeric regions for Nanopore sequencing
Supplementary Table 4. Benchmarking of draft assemblies for BB12 against IT reference genome
Supplementary Table 5. SV classes identified by each pipeline
Supplementary Table 6. Primers and probes used in duplex qPCR assay

Supplementary Table 1. Primers and Probes used in Duplex qPCR Assay used to quantify human and *P. falciparum* DNA quantities

**Species**	**Primer/Probe**	**Sequence detail (5' to 3')**	**Reference**
P. falciparum	Fal_F	TATTGCTTTTGAGAGGTTTTGTTACTTTG (GeneWorks, Australia)	Rosanas-Urgell *et al.*, 2010
	Fal_R	ACCTCTGACATCTGAATACGAATGC (GeneWorks, Australia)	
	Fal_P	FAM- ACGGGTAGTCATGATTGAGTT - MGB-NI (Cat No.4316033, Applied Biosystem, USA)	
Human	Hu_F	CTTACCACATCCGCTCCATC (GeneWorks, Australia)	Pinheiro, M.M., *et al.*, 2015
	HU_R	TTCACACTCTCCGTCACATTG (GeneWorks, Australia)	
	HU_P	LC640 -CACATCCCCAGTGCCGAGTTAGA- BB MOLBIOL, Germany)	

Supplementary Table 2: SV classes identified by each pipeline

SV Class	*Sniffles*	*GRIDSS*	*Assemblytics*
Deletion	DEL[a]	DEL	Deletion, Repeat_contraction, Tandem_contraction
Insertion	INS[b]	INS	Insertion, Repeat_expansion
Duplication	DUP[c], INVDUP[d]	DUP	Tandem_expansion
Inversion	INV[e], INVDUP	INV	
Translocation	BND[f]	CTX[g]	

a.deletion, b. insertion, c. duplication, d. inverted duplication, e. inversion, f. breakend, g. complex translocation.

Supplementary Table 3. Summary of flow cells and library preparation kits for successful sequencing.

Sample ID	Flow cell ID	Flow cell Version	Flow cell Chemistry	Library 1D Kit version	Barcode kit version	Volume loaded per flow cell (ul)	Total amount loaded per flow cell (ng)	Total amount loaded per flow cell (fmol)	Basecaller
BB12	FAF13833	MIN106	R.9.4	SQK-LSK108	NA	12	400	81	guppy
XHA	FAH05336	MIN107	R.9.5	SQK-LSK108	NA	12	350	71	dorado
3D7 mock infection (drWGA+ ethanol + 1V bead after end-prep)	FAF13999	MIN106	R.9.4	SQK-LSK108	EXP-NBD103 (NB01)	12	308	62	guppy
3D7 mock infection (drWGA+ ethanol + 0.45V bead after end-prep)	FAF13999	MIN106	R.9.4	SQK-LSK108	EXP-NBD103 (NB02)	12	308	62	guppy
3D7 mock infection (drWGA+ 1.8V bead + 1V bead after end-prep)	FAF13999	MIN106	R.9.4	SQK-LSK108	EXP-NBD103 (NB03)	12	308	62	guppy
3D7 mock infection (drWGA+ 1.8V bead + 0.45V bead after end-prep	FAF13999	MIN106	R.9.4	SQK-LSK108	EXP-NBD103 (NB04)	12	308	62	guppy
3D7 mock infection (un-WGA+ 1.8V bead + 1V bead after end-prep	FAF13999	MIN106	R.9.4	SQK-LSK108	EXP-NBD103 (NB05)	12	308	62	guppy
3D7 mock infection (un-WGA+ 1.8V bead + 0.45V bead after end-prep	FAF13999	MIN106	R.9.4	SQK-LSK108	EXP-NBD103 (NB06)	12	308	62	guppy
Cam_01	FAH44371	MIN107	R.9.5	SQK-LSK108	EXP-NBD103	12	390	79	dorado
Cam_02	FAH44371	MIN107	R.9.5	SQK-LSK108	EXP-NBD103	12	390	79	dorado
Cam_03	FAH44371	MIN107	R.9.5	SQK-LSK108	EXP-NBD103	12	390	79	dorado
PNG_05_1	FAH75903	MIN106	R.9.4	SQK-LSK108	EXP-NBD103	12	636	129	dorado
PNG_05_2	FAH71754	MIN106	R.9.4	SQK-LSK108	EXP-NBD103	12	556	112	dorado
PNG_05_3	FAH71754	MIN106	R.9.4	SQK-LSK108	EXP-NBD103	12	556	112	dorado
PNG_05_4	FAH50923	MIN107	R.9.5	SQK-LSK108	EXP-NBD103	12	612	124	dorado
PNG_05_5	FAH50923	MIN107	R.9.5	SQK-LSK108	EXP-NBD103	12	612	124	dorado
PNG_05_6	FAH50923	MIN107	R.9.5	SQK-LSK108	EXP-NBD103	12	612	124	dorado
PNG_05_11	FAH75903	MIN106	R.9.4	SQK-LSK108	EXP-NBD103	12	636	129	dorado
PNG_05_12	FAH71754	MIN106	R.9.4	SQK-LSK108	EXP-NBD103	12	556	112	dorado
PNG_05_14	FAH75903	MIN106	R.9.4	SQK-LSK108	EXP-NBD103	12	636	129	dorado
PNG_12_1	FAH55672	MIN106	R.9.4	SQK-LSK108	EXP-NBD103	12	396	80	dorado
PNG_12_2	FAH45235	MIN106	R.9.4	SQK-LSK108	EXP-NBD103	12	811	164	guppy

PNG_12_3	FAH79546	MIN106	R.9.4	SQK-LSK108	EXP-NBD103	12	480	97	guppy
PNG_12_4	FAH79546	MIN106	R.9.4	SQK-LSK108	EXP-NBD103	12	480	97	guppy
PNG_12_5	FAH79546	MIN106	R.9.4	SQK-LSK108	EXP-NBD103	12	480	97	guppy
PNG_16_1	FAF13829	MIN106	R.9.4	SQK-LSK108	EXP-NBD103	12	420	85	guppy
PNG_16_2	FAF13829	MIN106	R.9.4	SQK-LSK108	EXP-NBD103	12	420	85	guppy
PNG_16_3	FAH40814	MIN106	R.9.4	SQK-LSK108	EXP-NBD103	12	432	87	dorado
PNG_16_4	FAH40814	MIN106	R.9.4	SQK-LSK108	EXP-NBD103	12	432	87	dorado
PNG_16_5	FAH40814	MIN106	R.9.4	SQK-LSK108	EXP-NBD103	12	432	87	dorado
PNG_16_6	FAH44549	MIN107	R.9.5	SQK-LSK108	EXP-NBD103	12	696	141	dorado
PNG_16_7	FAH44549	MIN107	R.9.5	SQK-LSK108	EXP-NBD103	12	696	141	dorado
PNG_16_9	FAH44549	MIN107	R.9.5	SQK-LSK108	EXP-NBD103	12	696	141	dorado
PNG_16_10	FAH45235	MIN106	R.9.4	SQK-LSK108	EXP-NBD103	12	811	164	dorado

Supplementary Table 4. Nanopore sequencing output for different sample types.

A) Pure cultured *P. falciparum* laboratory isolates, XHA, BB12, B) 3D7 laboratory isolate mock infections, DNA from *P. falciparum* 3D7 spiked with human DNA, yellow highlight indicates optimal conditions, C) 33 field isolates from PNG and Cambodia. Colours indicate quality of data, green indicates data met quality threshold, blue indicates lower quality data did not meet sequencing threshold, red indicates low density samples that do not meet enrichment threshold. Note that thresholds are defined by sequencing outputs and provide guidance only. Lower density samples may produce better quality results with different enrichment methods such as sWGA. All raw data is available at European Nucleotide Archive, PRJEB101640 (field isolates) and PRJEB101641 (laboratory isolates).

A.

	Sequencing Output						Depth and Breadth of Coverage (Pf Genome)												Read Qualities		
Sample ID	Total bases (Gb)	Total reads	Mean read length (kb)	Median read length (kb)	Longest read length (kb)	Read length N50 (kb)	Mean depth of coverage	1x breadth of coverage	5x breadth of coverage	10x breadth of coverage	20x breadth of coverage	30x breadth of coverage	50x breadth of coverage	75x breadth of coverage	100x breadth of coverage	150x breadth of coverage	200x breadth of coverage	250x breadth of coverage	Proportion >Q5	Proportion >Q10	Proportion >Q15
XHA	3.42	388,561	8.80	4.58	261.32	14.63	120.30	99.38%	98.67%	98.21%	97.26%	96.51%	95.09%	92.88%	86.91%	5.38%	1.53%	0.95%	99%	76%	16%
BB12	2.07	298,286	6.95	3.44	157.32	15.26	76.06	99.55%	98.85%	98.08%	96.46%	94.63%	89.90%	54.85%	5.50%	1.54%	1.14%	0.70%	92%	69%	0%

B.

	Treatment			Sequencing Output										Read Qualities			Read Alignment	
	Digestion prior to WGA	Purification after T7 endonuclease treatment	Clean-up after end prep	Total bases (Gb)	Total reads	Median read length (kb)	Mean read length (kb)	Longest read length (kb)	1x breadth of coverage	5x breadth of coverage	10x breadth of coverage	20x breadth of coverage	30x breadth of coverage	Proportion >Q5	Proportion >Q10	Proportion >Q15	Proportion mapping to Pf	Proportion mapping to Human
3D7 Mock Infection	McrBC digestion	2.5V ethanol precipitation	1V bead purification	0.06	25292	1.73	2.55	34.64	75.97%	12.17%	0.72%	0.00%	0.00%	100%	96%	19%	83%	17%
	McrBC digestion	2.5V ethanol precipitation	0.45V bead purification	0.26	81719	2.31	3.13	49.85	96.76%	76.09%	36.56%	4.02%	0.00%	100%	96%	19%	84%	16%
	McrBC digestion	1.8V bead purification	1V bead purification	0.12	48950	1.63	2.43	41.98	91.00%	37.69%	5.77%	0.11%	0.00%	100%	95%	21%	87%	13%
	McrBC digestion	1.8V bead purification	0.45V bead purification	0.25	80543	3.12	2.29	44.61	96.69%	76.54%	37.17%	3.84%	0.00%	100%	96%	21%	85%	15%
	Undigested	1.8V bead purification	1V bead purification	0.21	79412	2.68	1.74	62.94	27.88%	0.29%	0.00%	0.00%	0.00%	100%	92%	5%	5%	95%
	Undigested	1.8V bead purification	0.45V bead purification	0.28	87727	3.24	2.36	57.00	30.28%	0.34%	0.00%	0.00%	0.00%	100%	94%	5%	4%	96%

C.

	Initial qPCR results	rWGA results				Sequencing Output						Depth and Breadth of Coverage (Pf Genome)						Read Qualities			Read Alignment				
Sample ID	Parasite density (copies/µL)	Av. parasite density (copies/µL)	St. dev. parasite density (copies/µL)	Av. fold enrichment	St. dev. fold enrichment	Total bases (Gb)	Total reads	Mean read length (kb)	Median read length (kb)	Longest read length (kb)	Read length N50 (kb)	Mean depth of coverage	1x breadth of coverage	5x breadth of coverage	10x breadth of coverage	20x breadth of coverage	30x breadth of coverage	Proportion >Q5	Proportion >Q10	Proportion >Q15	Proportion mapping to Pf	Proportion mapping to Pv	Proportion mapping to Human	Proportion unmapped	
PNG_16_1	27000	1.22E+10	6.79E+08	4.52E+05	2.51E+04	0.26	90,984	2.89	2.03	39.37	4.20	9.61	96.00%	75.80%	43.25%	8.52%	1.38%	100%	94%	20%	85%	0%	3%	12%	SELECT FOR SEQUENCING
PNG_12_1a	19500	1.03E+09	2.94E+08	5.29E+04	1.51E+04	0.10	40,235	2.60	1.83	54.75	3.72	2.32	76.10%	15.28%	1.29%	0.02%	0.00%	98%	17%	0%	52%	0%	35%	13%	
PNG_12_1	20864	7.62E+08	2.38E+07	3.65E+04	1.14E+03	0.87	447,858	1.95	1.44	48.52	2.75	16.43	94.27%	80.33%	64.52%	32.67%	15.36%	100%	39%	0%	44%	0%	40%	16%	
PNG_05_5	21000	4.98E+08	1.99E+08	2.37E+04	9.46E+03	2.23	1,176,980	1.89	1.41	155.47	2.54	30.01	97.67%	91.37%	81.44%	58.64%	38.76%	97%	10%	0%	31%	0%	52%	17%	
PNG_16_4	22800	4.84E+08	2.52E+08	2.12E+04	1.11E+04	0.05	13,435	4.00	2.72	83.04	5.46	1.11	52.22%	3.83%	0.11%	0.00%	0.00%	100%	54%	0%	48%	0%	40%	12%	
PNG_16_6	587	4.76E+08	4.24E+08	8.10E+05	7.22E+05	0.34	132,178	2.58	1.65	97.67	17.94	6.14	92.03%	55.81%	20.95%	1.84%	0.17%	99%	16%	0%	42%	0%	42%	16%	
PNG_05_4	16840	4.27E+08	3.74E+07	2.54E+04	5.58E+03	0.40	211,320	1.89	1.35	37.15	2.68	3.87	86.03%	33.14%	7.18%	0.40%	0.06%	98%	14%	0%	23%	0%	65%	13%	
PNG_16_7	1860	4.01E+08	4.03E+08	2.15E+05	2.17E+05	0.51	165,355	3.09	2.04	69.42	13.95	9.19	95.53%	73.76%	41.19%	7.39%	1.01%	98%	16%	0%	42%	0%	44%	14%	
PNG_16_3	13900	3.41E+08	2.96E+08	2.45E+04	2.13E+04	0.30	73,487	4.12	2.79	93.60	6.42	2.28	76.72%	14.72%	1.05%	0.00%	0.00%	100%	50%	0%	18%	0%	71%	11%	
PNG_16_5	1920	3.20E+08	3.60E+08	1.66E+05	1.88E+05	0.18	54,699	3.33	2.17	62.73	9.62	2.81	80.32%	21.41%	2.46%	0.04%	0.00%	100%	49%	0%	36%	0%	51%	13%	
PNG_05_3	20920	3.19E+08	2.96E+08	1.53E+04	1.42E+04	0.97	464,915	2.08	1.46	44.49	2.95	7.56	95.44%	65.39%	28.92%	4.17%	0.65%	100%	62%	0%	14%	0%	52%	34%	
PNG_16_2	32200	1.59E+08	1.53E+08	4.94E+03	4.77E+03	0.69	238,264	2.90	1.99	52.46	4.24	21.54	97.76%	91.24%	79.40%	47.46%	23.67%	100%	93%	17%	73%	0%	15%	13%	
PNG_05_7	12080	1.52E+08	7.48E+07	1.26E+04	6.19E+03	1.15	616,664	1.86	1.32	42.86	2.70	6.48	81.70%	48.82%	26.05%	4.67%	0.83%	100%	36%	0%	13%	0%	71%	16%	
PNG_05_6	21120	1.39E+08	4.13E+07	6.57E+03	1.96E+03	2.64	1,487,301	1.77	1.30	87.14	2.38	3.92	85.34%	33.28%	8.08%	0.58%	0.09%	98%	7%	0%	3%	0%	79%	18%	
PNG_05_2	12700	1.35E+08	1.21E+08	1.06E+04	9.49E+03	1.03	533,841	1.93	1.35	38.88	2.70	4.01	82.21%	32.53%	9.64%	1.20%	0.25%	100%	59%	0%	9%	0%	75%	16%	
PNG_16_9	15300	1.34E+08	1.95E+08	8.73E+03	1.27E+04	0.07	26,400	2.78	1.79	62.06	1.75	0.76	43.06%	1.60%	0.03%	0.00%	0.00%	99%	19%	0%	24%	0%	62%	14%	
PNG_05_8	1556	1.32E+08	1.50E+08	8.49E+04	9.66E+04	1.26	670,554	1.88	1.34	36.14	2.72	5.48	74.65%	39.97%	20.37%	4.10%	0.98%	100%	36%	0%	10%	0%	74%	16%	
PNG_12_2	7050	5.60E+07	3.26E+07	7.94E+03	4.63E+03	0.08	27,501	2.75	1.87	60.39	4.12	0.19	14.05%	0.09%	0.00%	0.00%	0.00%	98%	9%	0%	6%	0%	82%	12%	DO NOT SEQUENCE
PNG_05_9	2084	4.98E+07	1.50E+07	2.39E+04	7.18E+03	1.89	971,238	1.95	1.44	70.79	2.62	1.45	40.61%	7.77%	1.55%	0.05%	0.00%	100%	33%	0%	2%	0%	81%	17%	
PNG_05_10	8160	4.83E+07	4.52E+07	5.92E+03	5.54E+03	0.19	67,094	2.70	1.64	57.29	4.50	1.40	57.59%	6.90%	0.47%	0.00%	0.00%	100%	45%	2%	18%	0%	63%	20%	
Cam_01	960	2.09E+07	2.22E+07	2.18E+04	2.32E+04	0.25	125,243	2.03	1.40	37.54	1.11	3.23	68.47%	22.70%	8.00%	1.79%	0.58%	100%	44%	0%	30%	0%	35%	36%	
PNG_05_1	12560	1.94E+07	5.98E+06	1.55E+03	4.76E+02	0.57	339,590	1.68	1.21	34.30	2.42	4.27	72.59%	28.27%	10.90%	2.96%	1.25%	100%	31%	0%	17%	0%	59%	23%	
Cam_02	892	1.64E+07	9.99E+06	1.45E+04	1.68E+04	0.14	69,522	2.07	1.45	45.49	1.86	1.36	51.30%	7.36%	1.19%	0.10%	0.02%	100%	44%	0%	22%	0%	36%	43%	
PNG_16_8	2520	1.41E+07	2.12E+07	5.61E+03	8.40E+03	0.21	72,300	2.86	1.83	59.95	4.58	4.93	90.28%	46.51%	12.25%	0.58%	0.02%	100%	53%	0%	56%	0%	31%	13%	
PNG_05_11	916	8.91E+06	5.92E+06	9.73E+03	6.46E+03	0.51	294,020	1.75	1.23	49.38	2.57	0.32	21.54%	0.39%	0.02%	0.00%	0.00%	100%	34%	0%	1%	0%	81%	18%	
PNG_05_12	3000	1.05E+06	5.10E+05	3.49E+02	1.70E+02	0.69	384,101	1.78	1.78	55.98	2.62	0.19	13.70%	0.20%	0.01%	0.00%	0.00%	100%	66%	0%	1%	0%	86%	13%	
PNG_05_13	2340	2.66E+05	2.84E+05	1.14E+02	1.22E+02	0.27	96,700	2.78	1.78	62.29	4.44	0.73	39.19%	1.83%	0.08%	0.00%	0.00%	100%	48%	0%	6%	0%	79%	15%	
PNG_16_10	334	5.22E+06	3.45E+06	1.56E+04	1.03E+04	0.09	37,941	2.26	1.55	36.73	3.29	0.09	6.64%	0.05%	0.00%	0.00%	0.00%	98%	8%	0%	2%	0%	77%	20%	DO NOT ENRICH
PNG_05_14	276	1.75E+07	1.11E+07	6.35E+04	4.02E+04	0.46	328,600	1.41	0.99	29.91	2.09	2.17	63.13%	14.95%	3.34%	0.34%	0.07%	100%	35%	0%	11%	0%	68%	21%	
Cam_03	68	4.56E+06	5.21E+06	6.70E+04	7.66E+04	0.19	78,529	2.38	1.64	64.95	9.47	1.50	29.24%	8.29%	3.90%	1.38%	0.62%	100%	49%	0%	19%	0%	51%	31%	
PNG_12_3	23	1.56E+07	1.07E+07	6.77E+05	4.66E+05	1.34	671,992	1.99	1.38	64.53	3.01	0.70	41.67%	1.07%	0.02%	0.00%	0.00%	100%	33%	0%	1%	0%	86%	13%	
PNG_12_4	2.59	1.93E+04	3.12E+04	7.46E+03	1.20E+04	0.82	402,397	2.03	1.36	59.47	3.20	0.01	0.78%	0.02%	0.01%	0.00%	0.00%	100%	32%	0%	0%	0%	87%	13%	
PNG_12_5	0.674	1.67E+04	2.89E+04	2.48E+04	4.28E+04	0.99	523,996	1.90	1.32	65.81	2.81	0.12	8.01%	0.13%	0.01%	0.00%	0.00%	100%	32%	0%	0%	0%	85%	15%	

Supplementary Table 5. SNV Genotyping Concordance Rates in Homopolymeric Regions for Nanopore sequencing.

Sample: XHA (117X coverage)

	Genotype	*Unfiltered*	*Filtered*
Homopolymer <=6bp	Concordant	683731	676846
	Discordant	1649	507
Homopolymer >6bp	Concordant	7823	7641
	Discordant	106	19
Discordance in homopolymers <=6bp:		0.24%	0.08%
Discordance in homopolymers >6bp		1.34%	0.25%

Sample: BB12 (84X coverage)

	Genotype	*Unfiltered*	*Filtered*
Homopolymer <=6bp	Concordant	656892	649945
	Discordant	1179	399
Homopolymer >6bp	Concordant	7560	7475
	Discordant	99	54
Discordance in homopolymers <=6bp:		0.18%	0.06%
Discordance in homopolymers >6bp		1.29%	0.72%

Sample: Cam_01 (4.8X coverage)

	Genotype	*Unfiltered*	*Filtered*
Homopolymer <=6bp	Concordant	451550	303559
	Discordant	3528	529
Homopolymer >6bp	Concordant	5076	3170
	Discordant	161	27
Discordance in homopolymers <=6bp:		0.78%	0.17%
Discordance in homopolymers >6bp		3.07%	0.84%

Sample: Cam_02 (3X coverage)

	Genotype	*Unfiltered*	*Filtered*
Homopolymer <=6bp	Concordant	329574	169799
	Discordant	3083	282
Homopolymer >6bp	Concordant	3628	1753
	Discordant	146	23
Discordance in homopolymers <=6bp:		0.93%	0.17%
Discordance in homopolymers >6bp		3.87%	1.30%

Supplementary Table 6: Benchmarking of draft assemblies for BB12 against IT reference genome

	Illumina only (SPAdes)	Nanopore only (Flye)	Nanopore only polished (Flye + Racon (x10) + Medaka)	Hybrid Assembly
**No. contigs**	7916	26	17	17
**Total length (bp)**	22,078,011	23,219,961	23,291,451	23,327,354
**Largest contig (bp)**	122,827	3,357,453	3,368,580	3,374,248
**N50 length (bp)**	4192	1,618,402	1,619,698	1,621,841
**GC content (%)**	19.14	19.63	19.53	19.49
**Genome fraction (%)**	89.5	99.2	99.2	99.2
**Total aligned length**	20891182	23,101,799	23,178,139	23,213,577
**Mismatches (per 100kbp)**	61.14	12.21	16.87	13.50
**Indels (per 100 kbp)**	27.82	414.86	305.70	191.29
**BUSCO (Completeness)**	NA	C:98.8%[S:98.3%,D:0.5%], F:0.1%, M:1.1%, n:3642, E:1.8%	C:98.8% [S:98.3%, D:0.5%], F:0.1%, M:1.1%, n:3642, E:1.3%	C:98.7% [S:98.2%, D:0.5%], F:0.2%, M:1.1%, n:3642, E:0.8%

C = Complete BUSCOs, S = Complete and single-copy BUSCOs, D = Complete and duplicated BUSCOs, F = Fragmented BUSCOs, M = Missing BUSCOs, n = number of Benchmarking Universal Single-Copy Orthologs (BUSCOs), E = internal stop codons
